# Distinct regulation of Wnt signaling and EPZ5676 governs human UiPSM reprogramming via the CDX2/T/TBX6 core axis

**DOI:** 10.1186/s13619-026-00302-z

**Published:** 2026-08-24

**Authors:** Kaipeng Wang, Xingnan Huang, Huiru Gao, Junyang Li, Tao Huang, Minjing Ke, Yu Fu, Zejie Lin, Duanqing Pei, Yue Qin

**Affiliations:** 1 Fudan University, Shanghai, China; 2 Laboratory of Cell Fate Control, School of Life Sciences, Westlake University, Hangzhou, China; 3 Institute of Biology, Westlake Institute for Advanced Study, Hangzhou, China

**Keywords:** UiPSM, Wnt/β-catenin signaling, Reprogramming, Chromatin remodeling, *CDX2*/*T*/*TBX6* axis

## Abstract

An ideal in vitro system to study somitogenesis is induced presomitic mesoderm (UiPSM); the regulatory pathways of this system are not well understood. Here, integrated multi-omic and functional analyses were performed to elucidate the roles of Wnt signaling and EPZ5676 in UiPSM reprogramming. The findings showed that the Wnt/β-catenin signaling activation by CHIR is essential to induce UiPSM, which facilitates chromatin remodeling and the activation of major presomitic mesoderm (PSM) transcription factors (TFs). Conversely, EPZ5676 controls when reprogramming occurs and increases the efficiency of reprogramming by controlling chromatin dynamics without dictating cell fate. The TFs CDX2, T (Brachyury), and TBX6 were found to be a central regulatory axis whose functions were partially redundant. Mechanistically, CDX2 directly interacts with the regulatory regions of WNT3A, T, and TBX6, maintaining their chromatin accessibility and transcriptional activity and creating a positive feedback loop of Wnt/β-catenin-dependence. Moreover, the interaction between CDX2, T, and TBX6 with Wnt signaling is reciprocal to establish a stable regulatory network that preserves UiPSM identity and stemness. Taken together, this work shows that Wnt/β-catenin signaling coordinates UiPSM reprogramming and maintenance via the CDX2/T/TBX6 axis, which offers new mechanistic understanding of human mesoderm lineage specification and somatic cell reprogramming.

**Supplementary Information:**

The online version contains supplementary material available at 10.1186/s13619-026-00302-z.

## Background

The mesodermal lineage specification is a basic mechanism in the embryonic development of multicellular organisms (Aulehla and Pourquie 2009). The axial elongation and somitogenesis are directly mediated by the coordinated differentiation and rhythmic segmentation of the presomitic mesoderm (PSM), which forms the cellular basis of skeletal muscle, cartilage, vertebrae, and other tissues (Henrique et al. 2015; Saito and Suzuki 2020). Human PSM development dysregulation may lead to numerous congenital disorders, such as congenital scoliosis and vertebral malformations (Eckalbar et al. 2012; Samarkhanova et al. 2025; Sparrow et al. 2013). Nevertheless, due to ethical constraints related to the use of human embryos and species-specific variations between rodent models and human PSM development, the exact molecular pathways that control human mesoderm progenitor fate determination and somite formation are not fully understood.

Somatic cell reprogramming can be used to produce human urine-derived induced PSM progenitor cells (UiPSM) in vitro (Qin et al. 2022). These cells have self-renewal ability and directed differentiation potential, thus offering a new in vitro model of human somitogenesis (Gao et al. 2024; Qin et al. 2022) and disease modeling and regenerative medicine. However, the regulatory processes that govern UiPSM reprogramming are not fully comprehended. Despite Wnt/β-catenin signaling being a canonical mesoderm-inducing pathway, its specific regulatory contribution during UiPSM reprogramming, especially in the coordination of genome-wide chromatin remodeling dynamics, remains to be completely understood. Moreover, the fundamental transcriptional network that regulates UiPSM fate determination is not well-defined. In addition to transcriptional regulation, somatic cell reprogramming is fundamentally reliant on widespread epigenetic reconfiguration, but the role of major epigenetic modifiers in this process has not been systematically studied. As an illustration, the contribution of the histone methyltransferase Dot1L, which is a key regulator of chromatin state and cell fate determination, to UiPSM induction is unexplored. Therefore, specific small-molecule interventions, including the Dot1L inhibitor EPZ5676, offer a useful approach to unravelling these epigenetic dependencies. These knowledge gaps need to be systematically addressed to optimize the UiPSM induction system and fully explain its developmental path.

Past research has shown that transcription factors (TFs), such as *CDX2, T* (Brachyury), and *TBX6*, are required in the development of PSM in mice (Gaunt et al. 2008; Koch et al. 2017; van den Akker et al. 2002; Yamaguchi et al. 1999; Zhu and Lohnes 2022). Nevertheless, their functional roles, interaction networks, and regulatory responses to Wnt signaling in human UiPSM are yet to be fully defined. Moreover, reprogramming of chromatin accessibility is a major feature of cell fate changes, but there is limited direct multi-omic data to show that Wnt signaling induces PSM-specific signature genes by regulating chromatin accessibility.

To answer these scientific questions, this paper systematically examined the unique functions of CHIR-stimulated Wnt/β-catenin signaling and EPZ5676, a Dot1L inhibitor, in UiPSM reprogramming and found the *CDX2*/*Brachyury*(*T*)/*TBX6* axis to be a central regulatory unit of Wnt signaling. We have clarified the molecular pathway through which CDX2 directly interacts with the regulatory regions of *WNT3A*, *T*, and *TBX6* to regulate chromatin accessibility through integrative multi-omics analyses. Moreover, we showed that there is a Wnt/β-catenin -*CDX2/Brachyury (T)/TBX6* positive feedback loop that sustains the stemness and developmental potential of UiPSM. Collectively, this study delineates the key molecular mechanisms underlying UiPSM reprogramming and stemness maintenance. The results offer valuable information on the human mesodermal lineage specification and somitogenesis, thus enabling the creation of UiPSM-based applications in regenerative medicine and disease modeling. Collectively, this work delineates the key molecular mechanisms underlying UiPSM reprogramming and stemness maintenance. The results offer valuable information on the human mesodermal lineage specification and somitogenesis, thus enabling the creation of UiPSM-based applications in regenerative medicine and disease modeling.

## Results

### Distinct and essential roles of Wnt signaling and EPZ5676 in UiPSM reprogramming

**Fig. 1:**
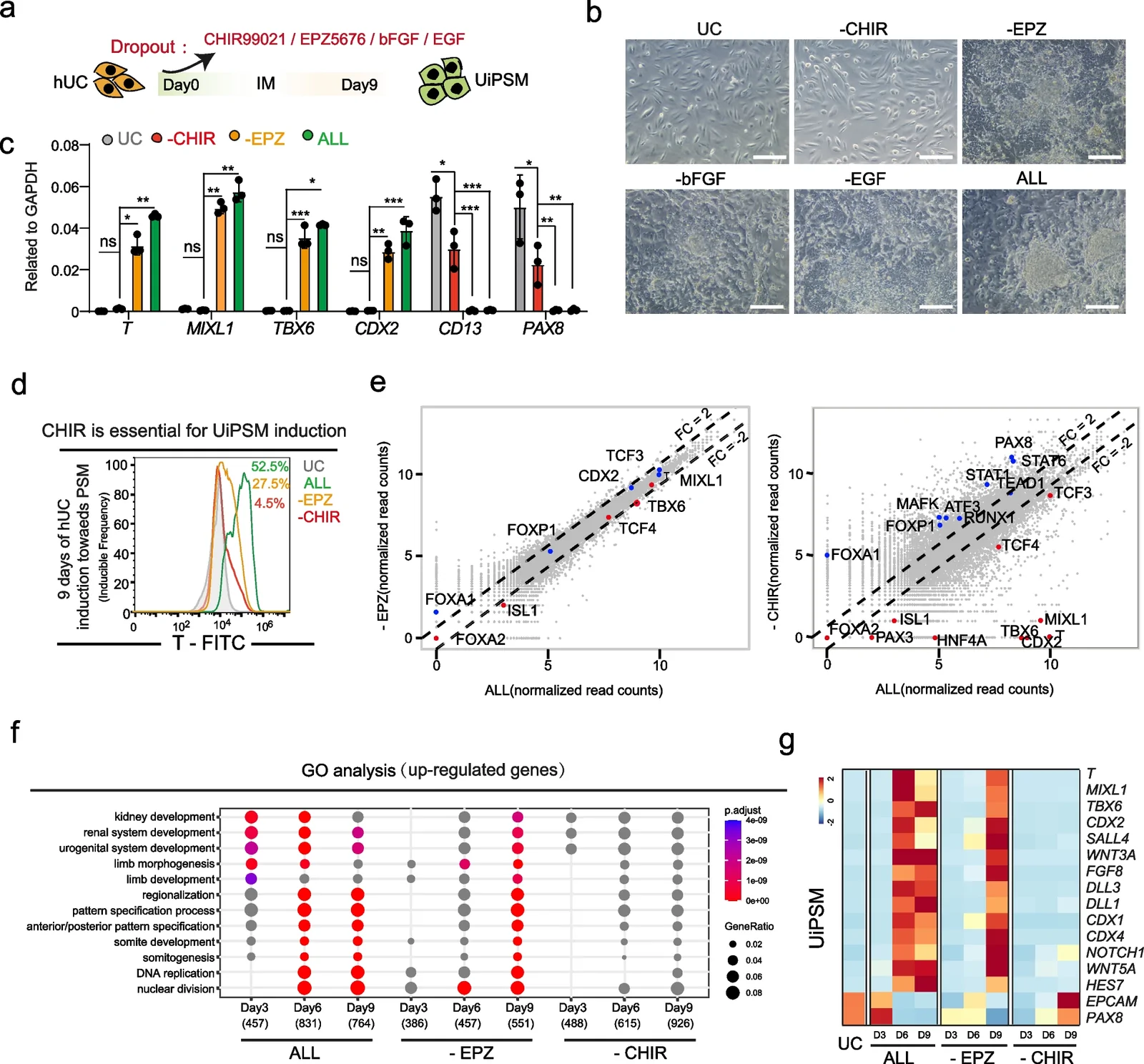
Dissecting the roles of CHIR99021 and EPZ5676 in UiPSM induction. **a** The schematic diagram shows the droupout of induction compounds during the UiPSM induction process. **b** Representative images show the effect of individual chemical generation of UiPSM. Scale bars, 100 µm. CHIR, CHIR99021; EPZ, EPZ5676; **c** qRT-PCR analysis of PSM and urine cell lineage markers(T, MIXL1, TBX6, CDX2 and PAX8, CD13) in hUC under indicated conditions. ALL (complete induction) robustly upregulates PSM markers; -CHIR (no CHIR99021) abolishes expression, confirming CHIR’s indispensability; -EPZ (no EPZ5676) only mildly reduces expression, supporting EPZ’s auxiliary role. *n* = 3, Data are mean ± SEM; unpaired t-test, two tail; *P < 0.05, **P < 0.01, ***P < 0.001; ns, not significant. **d** T (FITC) expression in hUC after 9-day reprogramming under indicated conditions. Values represent the frequency of T-positive (PSM) cells: 52.5% (complete induction,ALL), 27.5% (-EPZ), 4.5% (-CHIR), and baseline in uninduced UC. **e** Scatter plots of normalized RNA-seq read counts comparing (left) -EPZ vs. ALL and (right) -CHIR vs. ALL conditions. Dashed lines mark FC = ± 2. Core PSM transcription factors (red) are mildly reduced in -EPZ but severely downregulated in -CHIR. **f** GO analysis of upregulated genes at Day 3/6/9 under ALL (complete induction), -EPZ (no EPZ5676), and -CHIR (no CHIR99021) conditions. Bubble size = GeneRatio; color = p.adjust (deeper = more significant). **g** Heatmap of UiPSM/PSM gene expression under indicated conditions. Color scale: blue (low, z-score = − 2) to red (high, z-score = 2). ALL (complete induction) robustly upregulates PSM core genes at D9; -EPZ (no EPZ5676) shows mild reduction; -CHIR (no CHIR99021) abolishes expression, validating CHIR’s essential role and EPZ’s auxiliary function

Key components of the reprogramming culture conditions, including CHIR99021 (CHIR; a Wnt signaling activator), EPZ5676 (EPZ; a Dot1L inhibitor), basic fibroblast growth factor (bFGF), and epidermal growth factor (EGF), were experimentally manipulated to investigate the role of Wnt signaling in the UiPSM reprogramming system (Fig. 1a). CHIR99021 is a highly selective GSK-3 inhibitor that stabilizes cytoplasmic β-catenin by suppressing GSK-3 activity, thereby mimicking Wnt signaling (Chen et al. 2014; Huang et al. 2017). Withdrawal of CHIR completely abolished UiPSM colony formation. In addition, paraxial mesoderm (PSM) marker genes, including T, TBX6, MIXL1, and CDX2, failed to be upregulated, whereas urinary cell marker genes (PAX8 and CD13) remained highly expressed (Fig. 1c). In contrast, inhibition of either bFGF or EGF had no discernible effect on UiPSM reprogramming (Fig. 1b, c; S1). Furthermore, the absence of these factors did not redirect cell fate toward either the lateral mesoderm lineage, marked by HAND1 and ISL1, or the intermediate mesoderm lineage, marked by LHX1 and OSR1 (Fig. S1a). Collectively, these findings demonstrate that CHIR is indispensable for UiPSM reprogramming.

Moreover, induction efficiency following the removal of individual medium components was assessed by performing flow cytometric analysis of T-positive cells. CHIR withdrawal nearly abolished induction efficiency, whereas EPZ removal reduced the efficiency by approximately 50% (Fig. 1d). These results indicate that CHIR is indispensable for UiPSM induction, while EPZ exerts a comparatively moderate effect. To further investigate the molecular consequences of withdrawing these factors, RNA-seq was performed on day 9 (D9) of induction following CHIR or EPZ removal, and differential gene expression analyses were conducted. Compared with the ALL group, the EPZ-withdrawal group exhibited a strong correlation in gene expression profiles, with PSM signature genes largely distributed along the diagonal (Fig. 1e). In contrast, CHIR withdrawal caused a marked deviation in gene expression patterns, with PSM signature genes shifting substantially away from the diagonal (Fig. 1e). These results suggest that EPZ withdrawal has a limited effect on the overall transcriptional landscape, whereas CHIR removal induces profound transcriptional alterations.

To identify the critical temporal windows underlying the distinct effects of EPZ and CHIR withdrawal on induction efficiency, RNA-seq analyses were performed at multiple time points (days 0, 3, 6, and 9). Gene Ontology (GO) enrichment analysis revealed that biological processes associated with somite development, somitogenesis, and anterior–posterior patterning were significantly enriched on days 6 and 9 in the control PSM induction group (ALL) and on D9 in the EPZ-withdrawal group. In contrast, these processes were not enriched at any time point in the CHIR-withdrawal group (Fig. 1f). The formation of PSM requires a core gene regulatory network that governs these developmental processes. Among the key regulators, WNT3A, TBX6, FGF8, and CDX2 are essential for somitogenesis. In addition, DLL1 and NOTCH1, critical components of the Notch signaling pathway, regulate somite boundary formation and segmentation clock activity. HES7 promotes the periodic segmentation of somites, whereas MIXL1, CDX1, CDX4, SALL4, and WNT5A contribute to cell induction, differentiation, and maintenance of cellular identity. Notably, the expression of these representative genes was almost completely abolished following CHIR withdrawal (Fig. 1g). By contrast, EPZ withdrawal caused a pronounced delay in gene activation. Genes that were normally induced by day 6 (D6) in the ALL group were not activated until D9 in the absence of EPZ, accompanied by a substantial reduction in reprogramming efficiency (Fig. 1g). Collectively, these findings demonstrate that CHIR-mediated Wnt signaling is indispensable for UiPSM reprogramming, whereas EPZ5676 facilitates the timely induction and activation of PSM-associated genes.

### ATAC-seq profiling of CHIR- and EPZ-regulated chromatin dynamics in UiPSM reprogramming

**Fig. 2:**
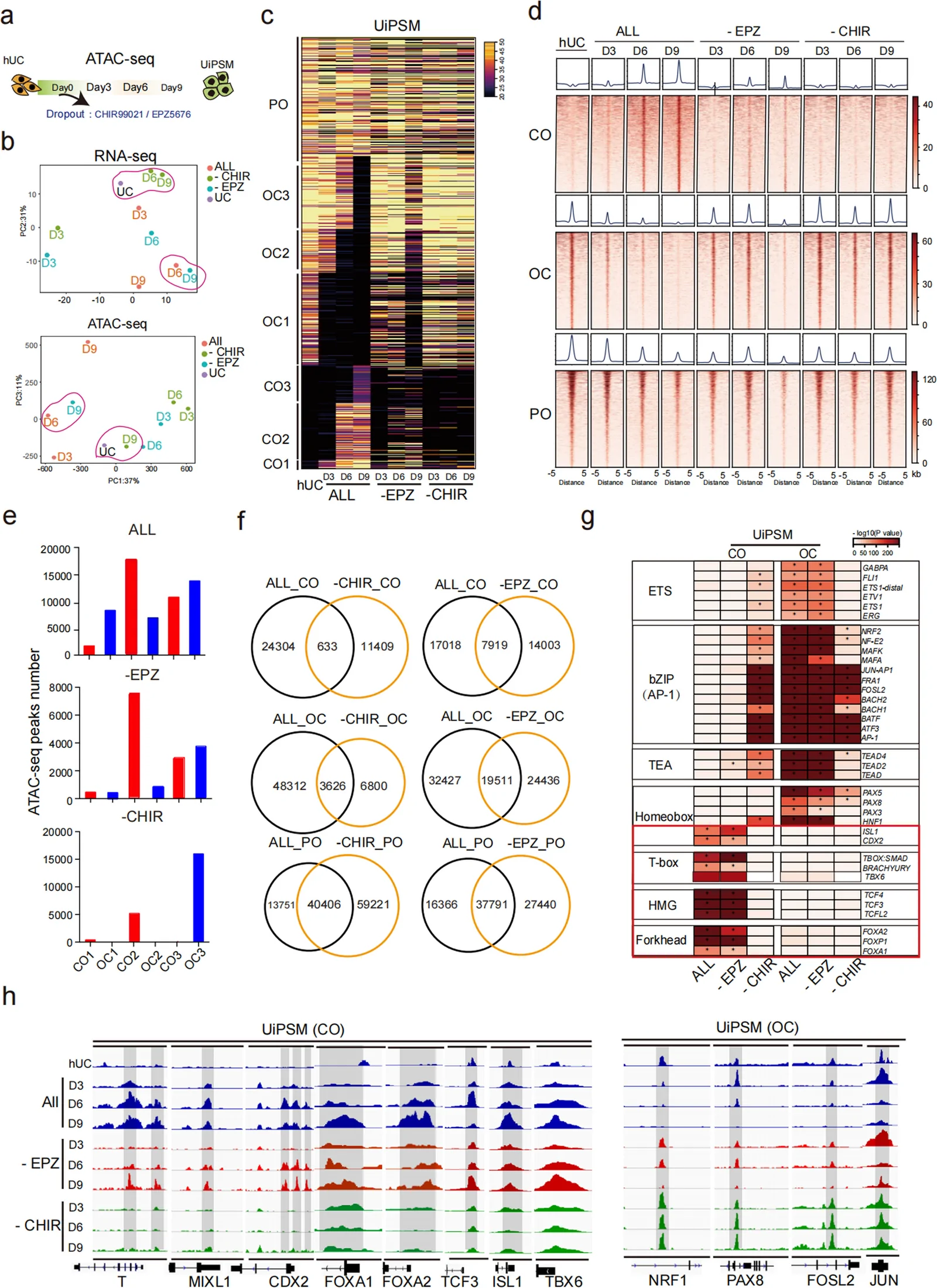
CHIR is essential role in chromatin accessibility remodeling. **a** Schematic of ATAC-seq experimental design. hUC were reprogrammed to UiPSM with ATAC-seq profiling at Day0/3/6/9, including CHIR99021/EPZ5676 dropout conditions to dissect chromatin accessibility dynamics. **b** PCA of transcriptomic and chromatin accessibility dynamics during UiPSM reprogramming.Top: RNA-seq PCA; Bottom: ATAC-seq PCA. Samples: ALL (complete induction), -CHIR (no CHIR99021), -EPZ (no EPZ5676), UC (uninduced) at D3/D6/D9. ALL/-EPZ samples cluster toward UiPSM over time, while -CHIR/UC remain distinct, validating CHIR’s essential role and EPZ’s auxiliary function. **c** Profiles and heatmaps showing signals of chromatin accessibility in UiPSM induction process with or without EPZ or without CHIR. closed in hUC but open in PSM state (CO), open in hUC but closed in PSM state (OC), permanently open in both initiating cells and PSM cells (PO). ALL (PSM state); Rows = regulatory modules; color scale: black (low, 20) to yellow (high, 50). **d** Integrative track and heatmap analysis of CO/OC/PO module activity during UiPSM reprogramming. Tracks (blue peaks) and heatmaps show signal intensity at UC, ALL (D3/D6/D9), -EPZ (D3/D6/D9), and -CHIR (D3/D6/D9). X-axis: genomic distance (kb); color scale: black (low) to red (high); **e** ATAC-seq peak counts for closed (C, red) and open (O, blue) regulatory modules under indicated conditions. ALL (complete induction) robustly accumulates open module (OC) peaks; -EPZ (no EPZ5676) shows mild reduction; -CHIR (no CHIR99021) abolishes OC peak activation, validating CHIR’s essential role in chromatin accessibility remodeling. **f** Venn diagrams of overlapping genomic regions in CO/OC/PO modules under indicated conditions. ALL (complete induction) overlaps far more with -EPZ (no EPZ5676) than with -CHIR (no CHIR99021). **g** TF motif enrichment heatmap for CO/OC modules under indicated conditions. Color scale: white (low, -log_10_(P) = 0) to dark red (high, -log_10_(P) = 200). CO module enriches PSM core TF motifs (red box: T-box, HMG, Forkhead) in ALL/-EPZ; OC module enriches AP-1/ETS/TEA motifs. -CHIR abolishes enrichment. **h** ATAC-seq signal tracks of CO/OC module gene loci under indicated conditions. ALL (complete induction, blue) and -EPZ (no EPZ5676, red) progressively increase chromatin accessibility at PSM core genes (CO) and decrease on OC module genes

Cellular functional states are largely determined by genome architecture. Numerous studies have employed ATAC-seq (Assay for Transposase-Accessible Chromatin using sequencing) to identify key regulatory factors involved in induced pluripotent stem cell (iPSC) reprogramming, thereby providing important insights into the underlying mechanisms of cellular reprogramming (Cao et al. 2018b; Li et al. 2017a; Yu et al. 2020). To identify critical downstream factors regulated by CHIR and EPZ during UiPSM reprogramming, ATAC-seq analysis was applied, as these two compounds were found to play essential roles in the reprogramming process (Fig. 2a). Principal component analysis (PCA) of the integrated ATAC-seq and RNA-seq datasets revealed that cells subjected to CHIR withdrawal on D9 clustered closely with human urinary-derived cells (UCs). In contrast, cells in the EPZ-withdrawal group on D9 exhibited profiles resembling those of the ALL group on D6 (Fig. 2b). These findings suggest that CHIR is indispensable for driving complete chromatin remodeling during UiPSM reprogramming. Conversely, EPZ depletion appears to arrest chromatin maturation at an intermediate stage of the reprogramming process.

Building on our previously established analytical model, which describes chromatin accessibility dynamics using a binary closed-to-open (CO) framework (Cao et al. 2018a; Li et al. 2017b), the roles of CHIR and EPZ in regulating chromatin accessibility during PSM induction were investigated. Our analyses revealed that PSM induction follows the same CO chromatin remodeling logic (Fig. 2c, d). Comparison of chromatin accessibility profiles between hUCs and PSM cells identified three distinct categories of chromatin loci: loci that were closed in hUCs and open in PSM cells (CO), loci that were open in hUCs and closed in PSM cells (OC), and loci that remained constitutively open in both cell types (PO). Notably, CHIR withdrawal disrupted these chromatin accessibility dynamics, preventing the proper reorganization of both OC and CO loci during reprogramming (Fig. 2c, d). In contrast, EPZ withdrawal largely preserved the overall CO chromatin remodeling pattern associated with PSM induction. However, it markedly reduced the intensity of chromatin accessibility signals and delayed the timing of chromatin-opening events (Fig. 2d). These findings suggest that CHIR is required for the establishment of appropriate chromatin remodeling programs, whereas EPZ primarily facilitates the efficiency and kinetics of chromatin accessibility changes during PSM reprogramming.

In particular, the CO and OC peaks were further classified into several subgroups based on the timing of chromatin opening and closing during the transition from hUCs to PSM cells (Fig. 2d). Peak quantification revealed that the number of OC peaks exceeded that of CO peaks on days 3 and 9 of UiPSM induction, whereas CO peaks were more abundant on D6 (Fig. 2e). Further quantitative analysis showed that, in the absence of EPZ or CHIR, approximately 20% and 60% of loci, respectively, failed to open properly, while nearly 30% and 90% failed to close correctly (Fig. 2f). These findings indicate that CHIR withdrawal severely disrupts chromatin opening and closing at critical loci, whereas EPZ withdrawal results in incomplete chromatin remodeling and delays the progression of these remodeling events.

To further elucidate the roles of CHIR and EPZ in chromatin dynamics, the transcription factor (TF) motifs enriched in open-to-closed (OC) and CO peaks under conditions with and without CHIR or EPZ treatment were compared, thereby identifying the DNA-binding proteins potentially involved in these processes (Fig. 2g). T-box, HMG, Forkhead, and specific Homeobox motifs were enriched at CO loci during UiPSM induction; however, these regions failed to open following CHIR withdrawal (Fig. 2g). This finding suggests that T-box, HMG, Forkhead, and Homeobox TFs play critical roles in establishing the Wnt-dependent chromatin accessibility landscape. In contrast, ETS, bZIP, and several Homeobox motifs were enriched at OC loci during UiPSM induction (Fig. 2g). Notably, CHIR withdrawal resulted in persistent accessibility defects at these regions, preventing normal chromatin closure (Fig. 2g). Taken together, these results suggest that Wnt signaling-regulated TF networks coordinate chromatin accessibility dynamics during UiPSM reprogramming.

Moreover, the chromatin accessibility dynamics of genes associated with motifs that failed to undergo chromatin opening following CHIR withdrawal were examined. ATAC-seq peaks within the regulatory regions of *MIXL1, CDX2, FOXA1, FOXA2, TCF3, ISL1,* and *TBX6* were nearly undetectable in human urinary-derived cells (UCs), emerged weakly on day 3 (D3), increased markedly by D6, and remained highly accessible on D9. This pattern reflects the progressive establishment and maintenance of chromatin accessibility during normal PSM specification. In contrast, peaks associated with *NRF1, PAX8, FOSL2,* and *JUN* exhibited reduced chromatin accessibility as early as D3 (Fig. 2h). As expected, loci that were programmed to become accessible showed limited accessibility at D3, gained substantial accessibility by D6, and ultimately reached high accessibility levels by D9. In contrast, loci destined for closure began to lose accessibility as early as D3 (Fig. 2h). Following CHIR withdrawal, however, loci that normally undergo chromatin opening failed to acquire accessibility throughout the reprogramming process, whereas loci that should have closed remained aberrantly accessible (Fig. 2h). Taken together, these findings indicate that CHIR is essential for both the activation and termination phases of PSM chromatin reprogramming, while EPZ serves as a critical modulator that fine-tunes the magnitude and temporal progression of chromatin accessibility changes.

### Wnt signaling controls UiPSM reprogramming through the T/CDX2/TBX6 Core Axis

To investigate how CHIR-stimulated Wnt signaling regulates chromatin accessibility dynamics, the TF motifs enriched within CO peaks were analyzed. These CO loci were enriched for binding motifs of the paraxial mesoderm-specific transcription factors (TFs) T (Brachyury), CDX2, and TBX6. This finding is consistent with previous studies demonstrating a direct binding-dependent mechanism underlying the activation of Wnt-responsive target genes (Yamaguchi et al. 1999).

**Fig. 3:**
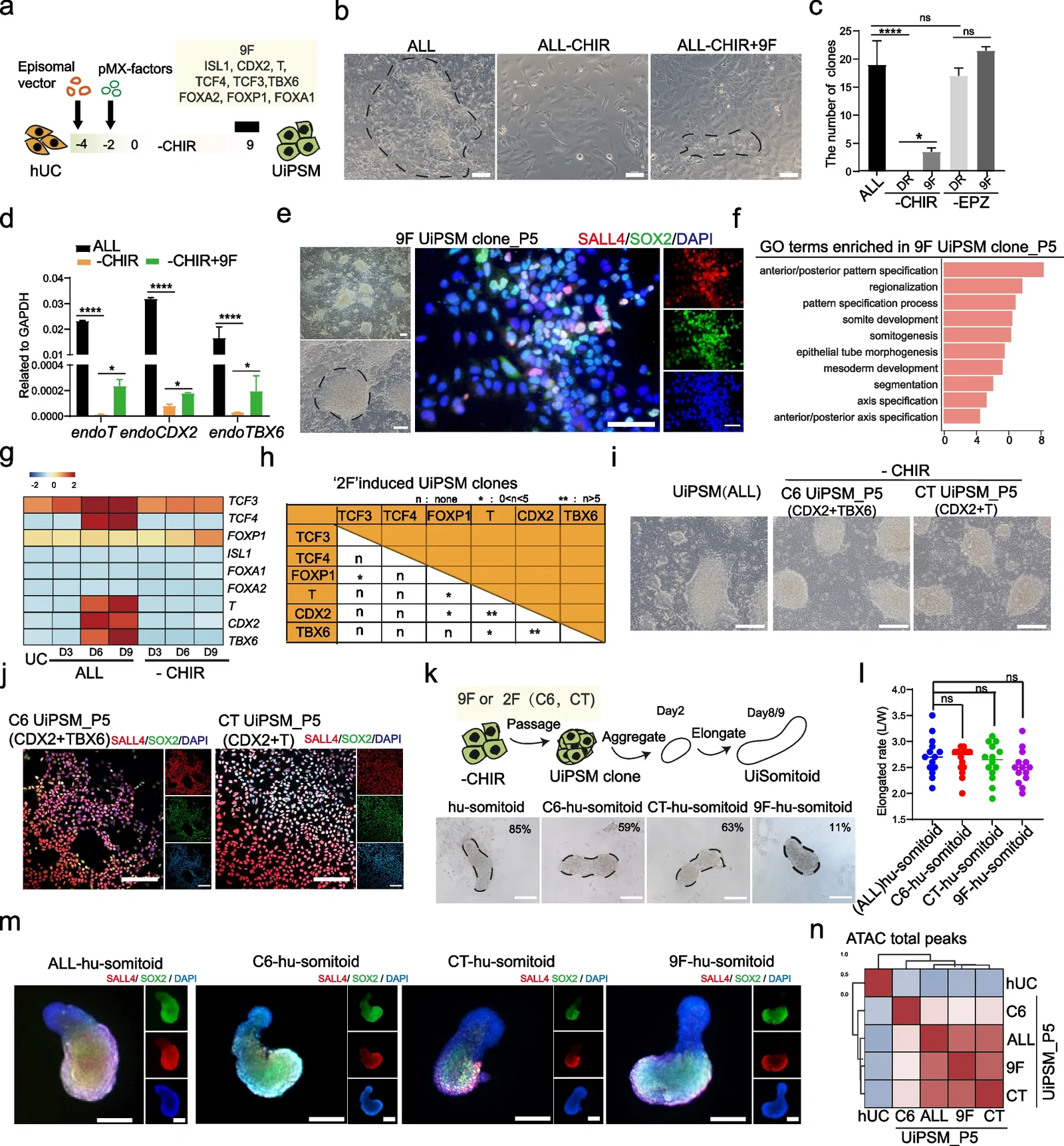
TF-Mediated UiPSM Reprogramming and Differentiation into UiSomitoids. **a** Schematic of TF overexpression-mediated UiPSM reprogramming. hUC were transfected with episomal and pMX vectors encoding 9 UiPSM core TFs (9F) at Day −4/−2, followed by 9-day induction (with/without CHIR99021) to test TF-driven UiPSM fate specification. **b** Representative images of UiPSM reprogramming under indicated conditions. ALL (complete induction) forms colonies (dashed circles); ALL-CHIR (no CHIR99021) abolishes colonies; ALL-CHIR + 9 F (9F overexpression) rescues colony formation. Scale bars, 100 µm. **c** Quantification of UiPSM colony numbers under indicated conditions. ALL (complete induction) shows the highest colony count; -CHIR (no CHIR99021) with 9 F overexpression (9F) rescues colony number vs. control (DR), while -EPZ (no EPZ5676) shows no significant difference between DR and 9F. *n* = 3. Data are mean ± SEM; unpaired t-test, two tail; *P < 0.05, ****P < 0.0001; ns, not significant. **d** qRT-PCR analysis of endogenous PSM core genes under indicated conditions. ALL (complete induction) robustly upregulates *endoT, endoCDX2, endoTBX6*; -CHIR (no CHIR99021) abolishes expression; -CHIR + 9 F (9F overexpression) restores expression. *n* = 3. Data are mean ± SEM;unpaired t-test, two tail; *P < 0.05, ****P < 0.0001. **e** Representative phase images of a 9F-induced inducible paraxial mesoderm (UiPSM) clone (passage 5, P5) derived from human urinary-derived cells (UCs). Left panels; Representative immunofluorescence (right) images staining for pluripotency markers SALL4 (red) and SOX2 (green), with DAPI (blue) as a nuclear counterstain. Right panels; Scale bars: 100 µm. **f** GO enrichment analysis of 9F-induced UiPSM clone (P5). Top enriched terms reflect PSM/developmental processes (somitogenesis, axis specification, mesoderm development), confirming stable maintenance of mesodermal lineage identity in passaged UiPSM clones. **g** Z-score heatmap of 9 UiPSM core TFs (9F) under indicated conditions. Color scale: blue (low, z-score = − 2) to red (high, z-score = 2). ALL (complete induction), ALL-CHIR (no CHIR99021) and ALL-CHIR + 9 F (9F overexpression). **h** 2F-mediated UiPSM clone formation efficiency matrix. Lower-triangular matrix of pairwise TF combinations (TCF3/4, FOXP1, T, CDX2, TBX6). Symbols: *n* = no clones; * = 0 < *n* < 5 clones; ** = *n* > 5 clones. CDX2-T/TBX6 combinations yield the highest clone counts (**), identifying synergistic TF interactions for UiPSM induction. **i** Representative images of 2F-induced UiPSM clones (P5). Left: original UiPSM; Middle: C6 (CDX2 + TBX6) UiPSM; Right: CT (CDX2 + T) UiPSM. Scale bars, 100 µm. **j** Immunofluorescence analysis of 2F-induced UiPSM clones (P5) upon withdraw CHIR. Left: C6 (CDX2 + TBX6) UiPSM; Right: CT (CDX2 + T) UiPSM. Staining: SALL4 (red)/SOX2 (green)/DAPI (blue). Scale bars: 100 µm (main). **k** Top: Schematic diagram illustrating the differentiation workflow from UiPSM clones (derived via 9 F or 2 F combinations: C6 = CDX2 + TBX6, CT = CDX2 + T, under -CHIR conditions) to UiSomitoids. Bottom: Representative phase-contrast images of human somitoids (hu-somitoids) derived from indicated UiPSM clones: hu-somitoid (control), C6-hu-somitoid (CDX2 + TBX6), CT-hu-somitoid (CDX2 + T), and 9F-hu-somitoid (9 core TFs). Formation efficiency labeled; Scale bars, 100 µm. **l** Quantification of hu-somitoid elongation rate (L/W) under indicated conditions. Scatter plot of control/C6(CDX2 + TBX6)/CT(CDX2 + T)/9F-hu-somitoids, hu-somitiod(ALL) as a control. *n* = 12. ns = no significant difference (one-way ANOVA). **m** Representative immunofluorescence images of human somitoid: hu-somitoid (control), C6-hu-somitoid (CDX2 + TBX6), CT-hu-somitoid (CDX2 + T), and 9F-hu-somitoid (9 core TFs). Cells are co-stained for pluripotency markers SALL4 (red) and SOX2 (green), with DAPI (blue) as a nuclear counterstain. Insets show single-channel staining for each marker. Scale bars: 100 µm. **n** ATAC-seq total peak correlation heatmap (hUC vs. UiPSM(ALL) and C6/ALL/9F/CT-UiPSM_P5 clones in CHIR-free condition. Color scale: blue (0) to red (1.0). Hierarchical clustering shows UiPSM_P5 clones cluster closely, distinct from hUC, validating similar chromatin accessibility profiles across TF-mediated UiPSM clones

Based on these observations, it was hypothesized that the nine TFs associated with CHIR-dependent peaks could functionally substitute for CHIR during UiPSM reprogramming (Fig. 3a). These factors, collectively termed 9 F, included *ISL1, CDX2, T, TCF3, TCF4, TBX6, FOXA2, FOXP1,* and *FOXA1* (Fig. 2g). It was found that overexpression of 9 F following CHIR withdrawal generated a limited number of colonies (Fig. 3b, c), which were subsequently designated as 9F-UiPSM. These colonies partially restored the endogenous expression of T, TBX6, and CDX2 (Fig. 3d) and could be passaged for at least five generations. Furthermore, 9F-UiPSM colonies at passage 5 (P5) maintained high expression levels of SALL4 and SOX2 (Fig. 3e), both of which are established markers of neuromesodermal progenitor (NMP) stemness in the posterior embryonic axis (Koch et al. 2017; Tahara et al. 2019). GO enrichment analysis identified somitogenesis and anterior–posterior axis specification as the most significantly enriched biological processes (Fig. 3f). These findings support the conclusion that 9 F functions downstream of Wnt signaling and can partially compensate for the loss of CHIR during UiPSM induction. In contrast, overexpression of 9 F following EPZ withdrawal did not affect the number of UiPSM colonies, indicating that 9 F does not mediate the regulatory effects of EPZ (Fig. 3c).

To determine whether the 9 F factors represent direct downstream effectors of Wnt signaling during UiPSM reprogramming, their expression patterns were first examined. Previous studies have shown that *FOXA1* and *FOXA2* play essential roles in endoderm development (Witman et al. 2023; Zhang et al. 2022). *ISL1* functions as a key regulator of cardiac differentiation within the lateral plate mesoderm (Witman et al. 2023), whereas *FOXP1* is involved in cardiac progenitor differentiation and heart morphogenesis (Zhang et al. 2022). *TCF3* serves as a central transcriptional regulator in the canonical Wnt signaling pathway, acting as either a transcriptional repressor or activator of downstream target genes and thereby coordinating gastrulation, primary axis formation, and head development (Spieker et al. 2004). Upon Wnt activation, β-catenin interacts with TCF4 to promote the expression of genes involved in somite boundary formation (Lin et al. 2006). RNA-seq analysis of UiPSM reprogramming revealed that the expression of *T, CDX2, TBX6,* and *TCF4* progressively increased following Wnt pathway activation. In contrast, *FOXA1, FOXA2,* and *ISL1* were expressed at relatively low levels throughout the process, whereas *TCF3* and *FOXP1* were constitutively expressed and showed no apparent regulation by Wnt signaling (Fig. 3g). These results suggest that *T, CDX2, TBX6,* and *TCF4* are more likely to function as direct Wnt-responsive factors during UiPSM reprogramming.

Each factor was subsequently overexpressed individually in CHIR-free induction medium, and the efficiency of UiPSM colony formation was evaluated. None of the single-factor treatments generated distinct clonal populations or significantly affected the expression of key PSM marker genes (Fig. S2a, b). A pairwise combinatorial screen using the six expressed factors (*T, CDX2, FOXP1, TCF4, TCF3,* and *TBX6*) was therefore performed to determine whether any combination could functionally replace CHIR during PSM induction. Several factor combinations produced a small number of colonies, including *T/CDX2, T/TBX6, T/FOXP1, CDX2/TBX6, CDX2/FOXP1,* and *TCF3/FOXP1* (Fig. 3h). Among these, the *T/CDX2* and *CDX2/TBX6* combinations exhibited the highest efficiency in promoting UiPSM induction (Fig. 3h). The resulting colonies, designated CT-UiPSM and C6-UiPSM, maintained stable morphology and continued to express high levels of *SOX2* and *SALL4* through P5 (Fig. 3i, j). Collectively, these findings suggest that Wnt signaling facilitates UiPSM reprogramming primarily through the activation of *T, CDX2,* and *TBX6*, which appear to serve as key downstream effectors of this pathway.

To further investigate the developmental potential of 9F-induced and 2F-induced UiPSM clones generated under CHIR-free conditions, their capacity to recapitulate the self-organization and elongation of human somitoids was evaluated. The 2F-induced clones were generated by overexpressing either T/CDX2 or CDX2/TBX6, whereas the 9F-induced clones expressed all nine candidate factors. Compared with the approximately 85% efficiency observed for standard UiPSM-derived human somitoids (hu-somitoids), the efficiencies of somitoid formation were reduced to 59% and 63% for the C6-hu-somitoid and CT-hu-somitoid groups, respectively. In contrast, the efficiency of the 9F-hu-somitoid group was markedly lower, reaching only 11% (Fig. 3k). Despite these differences in formation efficiency, statistical analysis revealed no significant differences in the rates of somitoid elongation among the resulting structures (Fig. 3l). Furthermore, TBX6 and SOX2 were localized to one end of the CT-hu-somitoid, C6-hu-somitoid, and 9F-hu-somitoid structures, corresponding to the posterior somite region (Fig. 3m). These findings indicate that *T, CDX2,* and *TBX6* are major downstream effectors of Wnt signaling during UiPSM reprogramming. The substantially lower efficiency observed in the 9 F condition further suggests that one or more of the additional factors may exert inhibitory effects on somitoid formation.

To further characterize chromatin dynamics during factor-mediated UiPSM reprogramming, ATAC-seq libraries were generated from human UCs, standard UiPSM clones (ALL), and factor-induced lines (CT, C6, and 9 F) established under CHIR-free conditions. Correlation analysis of genome-wide chromatin accessibility profiles revealed that the C6-, CT-, and 9F-induced UiPSM clones generated in the absence of CHIR were highly similar to standard ALL-UiPSM clones and were clearly distinct from the parental UCs (Fig. 3n, S5). These findings support the conclusion that *CDX2* + *T, CDX2* + *TBX6*, and the *9F* factor combination can partially substitute for CHIR by promoting chromatin remodeling and ultimately driving the formation of UiPSM clones.

To further investigate the individual contributions of the 9 F components to PSM induction, each factor from the CHIR-free UiPSM reprogramming system was systematically deleted. Deletion of *FOXP1, ISL1, FOXA1,* or *FOXA2* resulted in the formation of only a small number of colonies (Fig. S2c). In contrast, the simultaneous depletion of *FOXA1 and FOXA2* increased colony formation and enhanced the endogenous expression of *CDX2* and *FGF8* (Fig. S2d). Moreover, the combined deletion of *FOXA1* and *FOXA2* produced an additive effect, further improving reprogramming outcomes (Fig. S2e–g). These findings suggest that *FOXA1* and *FOXA2* exert inhibitory effects during UiPSM reprogramming, which may partially explain why reprogramming efficiency was lower under the 9 F condition than under CHIR-mediated or 2F-mediated induction. Taken together, the results indicate that although Wnt signaling activates chromatin regions enriched for motifs associated with the 9 F factors during UiPSM reprogramming, such activation alone is insufficient to induce the expression or function of all nine factors individually. These observations further highlight the complexity of Wnt-mediated regulation of chromatin dynamics during UiPSM induction and identify *T, TBX6,* and *CDX2* as key downstream regulators of this process.

### Wnt/β-catenin signaling and WNT3A positive feedback drive CHIR-dependent UiPSM reprogramming

CHIR activates canonical Wnt/β-catenin signaling by directly inhibiting GSK3β, thereby bypassing the requirement for exogenous Wnt ligands, although its activity may still depend on basal Wnt receptor signaling. GSK3β inhibition stabilizes active (dephosphorylated) β-catenin, promotes its translocation to the nucleus, and stimulates the expression of downstream Wnt target genes (Bain et al. 2007; Shah and Kazi 2022).

**Fig. 4:**
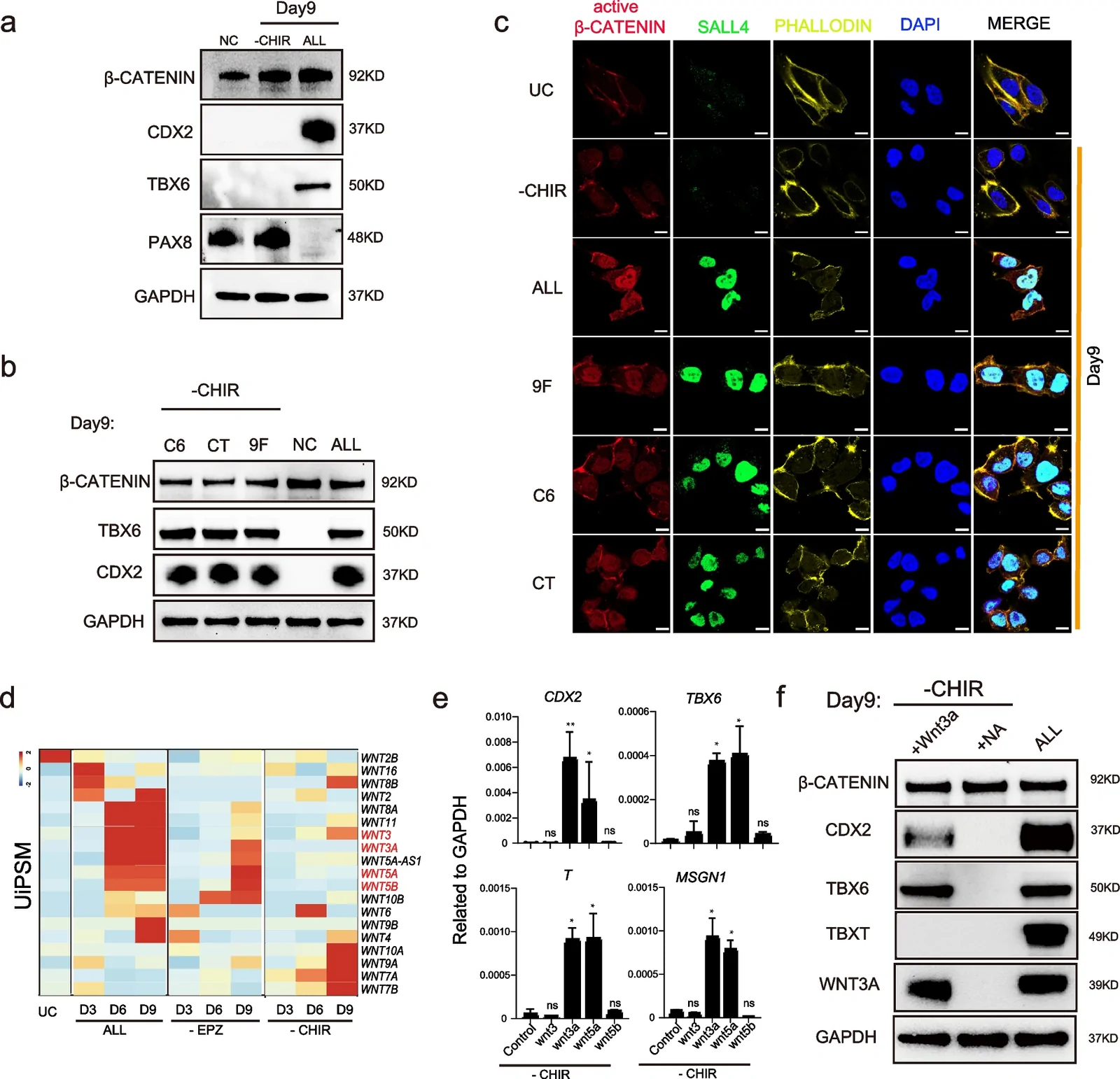
Wnt/β-catenin Signaling Drives PSM Specification in UiPSM Reprogramming. **a** Western blot analysis of Day 9 UiPSM reprogramming samples (NC/-CHIR/ALL). Proteins: total β-catenin (92 kDa), CDX2 (37 kDa), TBX6 (50 kDa), PAX8 (48 kDa); GAPDH (37 kDa) = loading control. **b** Western blot of Day 9 UiPSM reprogramming samples (Wnt-modulated groups: -CHIR background with C6, CT, 9 F, NC; ALL). Probed proteins: total β-catenin (92 kDa), TBX6 (50 kDa), CDX2 (37 kDa); GAPDH (37 kDa) as a loading control. **c** Immunofluorescence analysis of Day 9 UiPSM reprogramming samples (UC/-CHIR/ALL/9F/C6/CT clones). Staining: active β-catenin (red)/SALL4 (green)/Phalloidin (yellow)/DAPI (blue). Scale bars, 10 µm. **d** Z-score heatmap of WNT ligand genes during UiPSM reprogramming. Samples: UC/ALL(D3/D6/D9)/-EPZ(D3/D6/D9)/-CHIR(D3/D6/D9). Color scale: blue (−2) to red (2). **e** qRT-PCR analysis of PSM core genes (CDX2/TBXX6/T/MSGN1) at Day 9 (-CHIR conditions) under overexpressing WNT ligands (WNT3/WNT3/WNT5A/WNT5B). Expression normalized to GAPDH. Data: mean ± SEM; unpaired t-test, two tail; *P < 0.05, **P < 0.01; ns = not significant. **f** Western blot analysis of protein expression at Day9 of UiPSM reprogramming under -CHIR conditions (no CHIR99021): + Wnt3a (200 ng/mL), + NA (no ligand control), alongside positive control ALL (complete induction medium). Blots were probed for: total β-catenin (92 kDa), core paraxial mesoderm (PSM) proteins CDX2 (37 kDa), and TBX6 (50 kDa), WNT3A (39 kDa) with GAPDH (37 kDa) as a loading control

As discussed above, *β-catenin* regulates the expression of *CDX2* and *TBX6* during paraxial mesoderm specification (Yamaguchi et al. 1999; Zhao et al. 2014). Consistent with this role, *CDX2* and *TBX6* expression was absent in the untreated control (NC) and CHIR-withdrawn (-CHIR) groups at D9. In contrast, the urinary cell marker PAX8 remained expressed in these groups but was lost in the -ALL group (Fig. 4a). These observations indicate that CHIR-mediated activation of Wnt/β-catenin signaling is required for the acquisition of PSM identity while simultaneously suppressing urinary cell fate. Taken together, these findings suggest that Wnt/β-catenin signaling drives the transition from urinary cell identity to a PSM-like state and confirm that CHIR promotes UiPSM induction through direct activation of β-catenin signaling (Fig. 4a, c).

In our previous analysis of chromatin dynamics regulated by CHIR-activated Wnt signaling during UiPSM reprogramming, it was found that the 9 F and 2 F combinations (CT: CDX2 and T; C6: CDX2 and TBX6) could partially substitute for CHIR, thereby supporting reprogramming and the formation of UiPSM colonies. It was therefore investigated whether β-catenin signaling was also activated under these conditions. Our results showed that the C6, CT, and 9 F combinations effectively promoted UiPSM reprogramming in the absence of CHIR by increasing the accumulation of active β-catenin and simultaneously enhancing the expression of TBX6 and CDX2 (Fig. 4b). In addition, active β-catenin exhibited strong colocalization with SALL4 under all three conditions (Fig. 4c). These findings suggest that the 9 F and 2 F factor combinations can partially replace CHIR during UiPSM reprogramming by activating β-catenin signaling and reinforcing the expression of key PSM-associated transcription factors.

WNT3A is a direct downstream target of canonical Wnt/β-catenin signaling and participates in a positive feedback loop that amplifies pathway activity (Liu et al. 2022; Takano and Higuchi 2025). To identify Wnt ligands induced by CHIR-mediated activation of Wnt/β-catenin signaling during UiPSM induction, their expression following CHIR or EPZ withdrawal was examined. Several Wnt ligands, including WNT11, WNT8A, WNT3, WNT3A, WNT5A, and WNT5B, were upregulated in the PSM state. Among these, only WNT3A, WNT5A, and WNT5B remained highly expressed at D9 after EPZ withdrawal (Fig. 4d), suggesting that these ligands are particularly responsive to CHIR-induced Wnt signaling. Because WNT3 signaling strength influences the heterogeneity of NMP differentiation (Jin et al. 2025), whereas WNT5A and WNT5B regulate PSM cell migration and morphogenesis (Andre et al. 2015; Shi 2022), it was next sought to identify functionally relevant ligands by overexpressing WNT3, WNT3A, WNT5A, and WNT5B in the CHIR-withdrawn system. Overexpression experiments revealed that WNT3A and WNT5A partially compensated for the absence of CHIR by promoting the expression of PSM signature genes, including *CDX2, TBX6, T,* and *MSGN1*, with WNT3A exerting the stronger effect (Fig. 4e). To further evaluate whether WNT3A could functionally substitute for CHIR during UiPSM reprogramming, recombinant Wnt3a (+ Wnt3a) or a control treatment (+ NA) was added to CHIR-free cultures at D9. Active β-catenin and the PSM markers CDX2 and TBX6 were detected in both the Wnt3a-treated group and the positive control group but were absent in the negative control group. In contrast, total β-catenin levels did not differ significantly among the groups (Fig. 4f). These results indicate that WNT3A can partially restore Wnt/β-catenin signaling and PSM gene expression in the absence of CHIR; however, it cannot fully recapitulate the reprogramming effects of CHIR.

### CDX2 maintains UiPSM identity and stemness via a Wnt/β-catenin dependent regulatory network

**Fig. 5:**
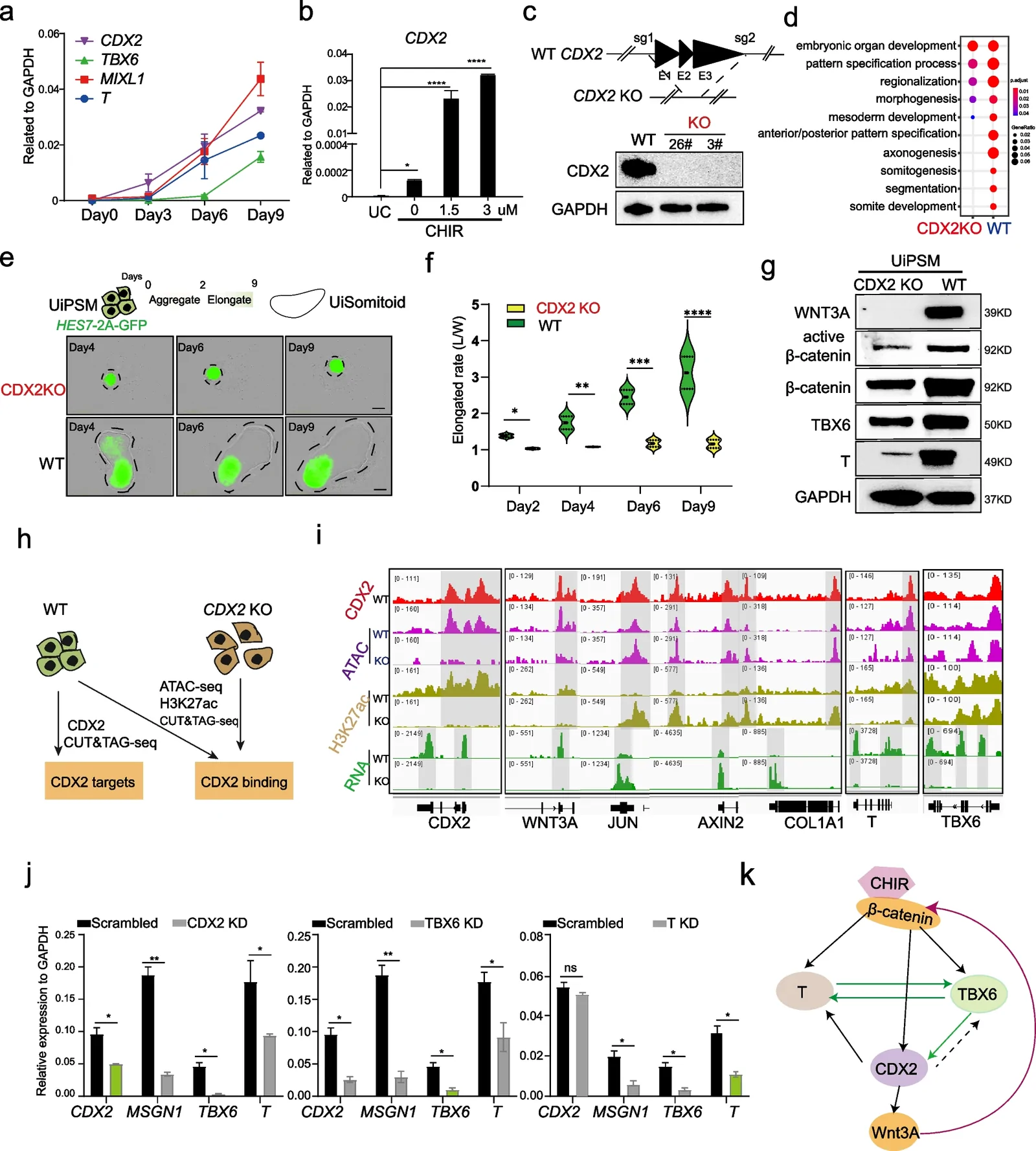
CDX2-Mediated Transcriptional and Chromatin Regulatory Networks Govern UiPSM Specification and UiSomitoid Morphogenesis. **a** Time-course qRT-PCR of PSM core genes (CDX2/TBX6/MIXL1/T) during UiPSM reprogramming (Day0/Day3/Day6/Day9). Expression normalized to GAPDH. *n* = 3. Error bars = mean ± SEM. **b** qRT-PCR analysis of CDX2 expression under CHIR99021 dose–response (UC/0/1.5/3 µM CHIR). Expression normalized to GAPDH. *n* = 3. Statistical significance: *P < 0.05, ****P < 0.0001. **c** Schematic of CRISPR-Cas9-mediated CDX2 gene knockout. Top: CRISPR-Cas9 strategy (sg1/sg2 target CDX2 exons 1/3, core region deletion). Bottom: Western blot of WT/26#/3# clones. CDX2 absent in KO clones; GAPDH = loading control. **d** Bubble plot comparing GO enrichment profiles between CDX2 KO (red) and WT (blue) UiPSM clones. Y-axis lists top GO terms. Bubble size = GeneRatio; color = p.adjust (red = 0.01 to blue = 0.04). **e** Top: Schematic illustrating the differentiation workflow from HES7-2A-GFP labeled UiPSM clones (derived under -CHIR conditions) to UiSomitoids. Bottom: Representative fluorescence images of HES7-2A-GFP (green, somitogenesis marker) in CDX2 KO (top row) and WT (bottom row) UiPSM clones at Day4, Day6, and Day9 of somitoid differentiation. Dashed lines highlight compact aggregate/elongated somitoid structures. Scale bars, 100 µm. **f** Violin plot of UiSomitoid elongation rate (L/W) across Day2/Day4/Day6/Day9 in CDX2 KO (yellow) and WT (green) clones. Statistical significance: *P < 0.05, **P < 0.01, ***P < 0.001, ****P < 0.0001 (one-way ANOVA). **g** Western blot analysis of CDX2 KO/WT UiPSM cells. Blots were probed for: WNT3A (39 kDa), active β-catenin (92 kDa), total β-catenin (92 kDa), TBX6 (50 kDa), T (49 kDa); GAPDH (37 kDa) as a loading control. **h** Multi-omic strategy to dissect CDX2-binding targets and regulatory networks in UiPSM: WT clones for identifying CDX2 target genes; WT + KO clones for mapping CDX2 binding sites and chromatin regulatory programs. **i** Multi-omic genome browser tracks (WT/CDX2 KO UiPSM clones) for key target genes (*CDX2/WNT3A/T/TBX6* etc.). Tracks: CDX2 binding (red), ATAC-seq (purple), H3K27ac (olive), RNA-seq (green). Gray = CDX2 regulatory regions. **j** qRT-PCR analysis of PSM core genes (CDX2/MSGN1/TBX6/T) under CDX2/TBX6/T KD conditions (Scrambled control). Expression normalized to GAPDH. *n* = 3. Data: mean ± SEM;unpaired t-test, two tail; *P < 0.05, **P < 0.01; ns = not significant. **k** Regulatory network model of Wnt/β-catenin and core PSM factors (T/TBX6/CDX2/Wnt3A) in UiPSM. CHIR activates β-catenin to induce PSM factors; CDX2/T/TBX6 reciprocally regulate each other; CDX2 → Wnt3A → β-catenin forms a positive feedback loop to sustain UiPSM fate

As described above, overexpression of CDX2/T or CDX2/TBX6 promotes UiPSM reprogramming by activating β-catenin signaling in the absence of CHIR. UiPSM cells can also be maintained in long-term culture under stable conditions, as previously reported (Qin et al. 2022). It was therefore investigated whether UiPSM maintenance relies on a similar mechanism, whereby CHIR activates downstream PSM-specific TFs through β-catenin signaling. Among these factors, CDX2 appeared to be a key regulator because its expression is induced early during reprogramming (D3 after CHIR treatment) and increases in a CHIR concentration-dependent manner (Fig. 5a, b). Based on these observations, the mechanistic role of CDX2 in the maintenance of cultured UiPSM cells was further explored.

To define the specific role of CDX2 in UiPSM cells and recapitulate somitogenesis, a CDX2-knockout (KO) UiPSM cell line was generated. Loss of CDX2 not only disrupted PSM identity but also impaired anterior–posterior axis elongation (Fig. 5c–f), consistent with the defective axial elongation observed in Cdx2-deficient mouse embryos (Savory et al. 2009). Similar results were obtained in independent CDX2 knockdown (KD) experiments, further demonstrating that loss of CDX2 function compromises UiPSM identity (Fig. S3a–c). Together, these findings establish CDX2 as a critical regulator of UiPSM developmental competence. The dependence of UiPSM stemness maintenance on Wnt/β-catenin signaling was next examined. Deletion of CDX2 resulted in a complete loss of WNT3A expression and a substantial reduction in both total and active β-catenin levels (Fig. 5g). Consistent with these changes, T expression was markedly decreased, whereas TBX6 expression was only moderately reduced (Fig. 5g). These observations suggest that WNT3A is a direct downstream target of CDX2 and that CDX2 also regulates the expression of T and TBX6.

To identify CDX2-binding sites and assess CDX2-dependent changes in chromatin accessibility, CUT&Tag and ATAC-seq analyses, respectively, were performed (Fig. 5h). Integrative multi-omics analyses revealed that CDX2 directly binds to regulatory elements of key developmental genes in UiPSM cells, including WNT3A, T, and TBX6. Following CRISPR-mediated knockout (KO) of CDX2, chromatin accessibility, as measured by ATAC-seq, and active enhancer marks, as indicated by H3K27ac enrichment, were markedly reduced at these loci. These changes were accompanied by an almost complete loss of *WNT3A* transcription, substantial downregulation of T, and reduced expression of TBX6 at the RNA level. In contrast, CTNNB1 (β-catenin) expression remained largely unchanged (Fig. S3d). Consistent with the loss of WNT3A, expression of AXIN2, a core negative regulator of Wnt/β-catenin signaling, was increased in CDX2-KO UiPSM cells. The expression of differentiation-associated genes, including *JUN* and *COL1A1*, was also elevated (Fig. 5i). Collectively, these findings indicate that CDX2 functions as a key upstream regulator that directly controls the chromatin accessibility and transcriptional activity of WNT3A and T, while indirectly regulating TBX6 to maintain the UiPSM developmental program. It was further examined whether CDX2 directly mediates the Wnt-dependent chromatin modifications of the nine factors identified above. Although CDX2 directly bound to the genomic loci of several genes, including *FOXA1, TCF3, TCF4, FOXP1, FOXA2,* and *ISL1*, its loss had minimal effects on the chromatin accessibility dynamics of these genes (Fig. S3d). These results suggest that Wnt signaling can regulate chromatin dynamics through mechanisms that are not strictly dependent on CDX2-mediated control of downstream target genes.

To elucidate the regulatory relationships among *CDX2, TBX6,* and* T*, each factor in UiPSM cells was individually knocked down. CDX2 KD directly affected the expression of both T and TBX6. Similarly, TBX6 KD altered the expression of CDX2 and T, whereas T KD primarily affected CDX2 expression. Notably, all three perturbations influenced the expression of MSGN1, a marker of PSM cells (Fig. 5j; S3a, e). Further analysis revealed that WNT3A, TBX6, and T are regulated by CDX2. In addition, TBX6 is regulated by both CDX2 and T, whereas T, in turn, regulates TBX6. Together, these interactions, combined with inputs from Wnt/β-catenin signaling, constitute a dynamically balanced regulatory network (Fig. 5k). This coordinated network, established through crosstalk between Wnt/β-catenin signaling and the TFs CDX2, T, and TBX6, is essential for maintaining proper cell fate determination and stemness in UiPSM cells.

### FGF signaling regulates UiPSM identity and morphogenesis

As noted above, FGF signaling plays a critical role in vertebrate segmentation (Turner et al. 2014; Wahl et al. 2007). It establishes a posterior-to-anterior gradient within the presomitic mesoderm (PSM), thereby regulating cell maturation and the positioning of segmental boundaries (Aulehla and Pourquie 2009; Qin et al. 2022; Turner et al. 2014). In addition, FGF signaling regulates segmentation clock oscillations during mouse somitogenesis (Wahl et al. 2007) through upstream modulation of the NOTCH and WNT pathways. However, removal of FGF2 from the UiPSM reprogramming system did not disrupt the reprogramming process (Fig. 1b–d). This observation may be explained by the endogenous production of FGF ligands by cultured cells in response to WNT activation (Diaz-Cuadros et al. 2020). To further investigate the role of FGF signaling, UiPSM cells were treated with the FGF agonists FGF8 and FGF4 or with the FGF receptor inhibitor PD173074 to evaluate their functional contributions (Fig. S4a). Treatment with FGF agonists had minimal effects on the expression of UiPSM marker genes and did not significantly affect anterior–posterior axis elongation (Fig. S4b–d). In contrast, low-dose treatment with PD173074 reduced the expression of UiPSM marker genes, including *TBX6, CDX2, FGF8,* and *DLL3*, and impaired axial elongation (Fig. S4e–g). These results indicate that exogenous FGF agonists are largely redundant, likely because endogenous FGF signaling is already sufficient to support UiPSM development. In contrast, inhibition of endogenous FGF signaling severely compromises UiPSM identity and developmental progression.

## Discussion

The current work logically elucidates the molecular pathways of urine cell-induced PSM (UiPSM) reprogramming and stemness maintenance, thereby establishing a hierarchical regulatory network that revolves around Wnt/β-catenin signaling and the CDX2/T/TBX6 core axis. Herein, we discuss the key findings, their implications for developmental biology and regenerative medicine, and how they relate to the prior studies. Notably, the molecular regulatory cascades described above are supported by multi-omic profiling and small-molecule intervention results, which mainly demonstrate correlative molecular interactions. Definitive causal regulatory relationships remain to be further validated via gene knockout and overexpression functional assays.

Our results demonstrate that CHIR- activated Wnt/β-catenin signaling is essential to induce UiPSM, with its deletion fully blocking PSM fate conversion. Conversely, the Dot1L inhibitor EPZ5676 stimulates chromatin maturation and allows the transition to the subsequent reprogramming phase. One of the key discoveries of the study is the discovery of the CDX2/T/TBX6 axis as a minimal functional module downstream of Wnt/β-catenin signaling that mediates UiPSM reprogramming. Although overexpression of the nine-factor panel (9F) partially restores UiPSM reprogramming in the absence of CHIR, its induction efficiency is grossly impaired. Conversely, simplified dual combinations (CDX2/T and CDX2/TBX6) have strong reprogramming potential and can effectively replace CHIR activity. The decreased efficiency of the full-factor cocktail is probably due to the inhibitory activity of FOXA1 and FOXA2 in the 9 F panel, indicating that the CDX2/T/TBX6 tripartite module is the most basic downstream effector of Wnt signaling. These results are in line with classical experiments in mouse embryos that have shown that CDX2, T (Brachyury), and TBX6 are essential in the development of PSM and somitogenesis. Importantly, our study further validates their conserved functions in human somatic cell reprogramming, thereby linking evolutionary conservation to translational relevance.

The UiPSM system in itself is a major breakthrough in the study of human somitogenesis and vertebral malformations. In contrast to animal models or pluripotent stem cell (PSC)-derived PSM systems (Ikeya et al. 2019; Sanaki-Matsumiya et al. 2022; Xi et al. 2017; Yamanaka et al. 2022), UiPSM can be produced using easily accessible and non-invasive urine-derived cells, allowing patient-specific disease modeling. Since there are known links between CDX2 or TBX6 mutations and congenital scoliosis and vertebral malformations, UiPSM is an excellent model to study the molecular pathways of these diseases in a human cellular system. Moreover, the capacity of UiPSM cells to develop into skeletal muscle cells, osteoblasts, and chondrocytes, along with their stable growth potential, further increases their translational potential in regenerative medicine applications to repair mesoderm-derived tissues.

Although these advances have been made, there are a number of limitations that must be considered. To begin with, the downstream epigenetic effects of EPZ5676 in UiPSM reprogramming are not well understood. Future research ought to determine the Dot1L-mediated histone modifications that control the temporal expression of PSM-associated genes. Second, the individual roles of each factor in the 9 F panel have not been completely defined and the pathways through which CHIR controls chromatin accessibility at these loci are not well understood. Third, other Wnt signaling pathways, including the planar cell polarity pathway, should be considered in UiPSM reprogramming, which is especially relevant considering that they have been reported to be linked with vertebral malformations (Hayes et al. 2014).

Finally, our results indicate that Wnt/β-catenin signaling coordinates UiPSM reprogramming by chromatin remodeling and CDX2/T/TBX6 core regulatory axis activation, and a WNT3A-positive feedback loop helps to maintain stemness. The paper contributes to the knowledge of human mesodermal lineage specification, offers a simplified approach to UiPSM induction, and further establishes UiPSM as a powerful platform to study developmental biology and translational research. This regulatory framework will be extended in future studies that use UiPSM to simulate vertebral malformations or to optimize regenerative therapies. Accordingly, we only objectively characterize the correlative regulatory network in this work and avoid drawing absolute causal conclusions based solely on current data.

## Methods

### UiPSMs induction

As described previously (Qin et al. 2022), 1.5 × 10⁶ to 2.0 × 10⁶ urine-derived cells at passage 1 or 2 were prepared. The cells were electroporated using the Amaxa™ Basic Nucleofector™ Kit for Primary Mammalian Epithelial Cells (Lonza) with the non-integrating plasmids pEP4EO2SET2K and pCEP4-miR-302–367. Subsequently, the transduced cells were seeded onto Matrigel-coated 12-well plates at a splitting ratio of 1:6 to 1:8 and cultured in REGM medium for 3–6 days until reaching 60% confluence. At this stage, induction medium (IM) was added. The medium was refreshed every two days for 8–12 days. The IM comprised advanced DMEM/F12 supplemented with 3 µM CHIR99021, 10 ng/ml bFGF, 5 ng/ml EGF, and 5 µM EPZ5676. For UiPSM maintenance, cells were cultured in advanced DMEM/F12 supplemented with 3 µM CHIR99021, 10 ng/ml bFGF, 5 ng/ml EGF. UiPSMs were passaged every 5 days by digestion with 0.25% Trypsin–EDTA (Gibco) and re-seeded at a density of 1 × 10^5^ cells per well onto Matrigel-coated 12-well plates in maintenance medium.

### UiPSMs self-organized into hu-somitoids

We obtained approximately 300 UiPSM cells and treated them with CHIR99021 (CHIR), a potent agonist of the Wnt/β-catenin signaling pathway. The initial treatment lasted 48–72 h, followed by an extended culture period of 5 to 6 days to generate human somitoids (hu-somitoids).

### Plasmid construction and virus production

In this report, the full-length coding sequences of individual factors—*FOXA1, FOXA2, FOXP1*, *ISL1, T, TBX6, CDX2, TCF3, TCF4, WNT3, WNT3A, WNT5A, WNT5B*, *JUN* and *FOSL2—*were amplified from UiPSM and human embryonic stem cell (hESC) cDNA via PCR. The resulting amplicons were cloned into the pMXs expression vector. Short hairpin RNAs (shRNAs) were cloned downstream of the human U6 promoter in PLKO.1 vector. Specifically for *CDX2*, *T,* and *TBX6* knockdown, four shRNAs targeting the coding sequences (CDS) of these genes were designed (Supplementary Table 1).

For retroviral and lentiviral production, HEK293T cells were transfected with individual plasmids alongside the retroviral packaging vector (pCL) or lentiviral packaging vector (pSPAX2 and PMD.2G) using PEI. 48 h post-transfection, viral supernatants were collected via syringe and filtered through 0.45-µm filters.

To evaluate the effects of these factors on UiPSM reprogramming in the absence of CHIR99021, human urine-derived cells were seeded at a density of 5 × 10^4^ cells per well in 12-well plates containing 293 T medium (DMEM supplemented with 10% Fetal bovine serum). Cells were infected with retroviruses for 24 h and lentiviruses for 8 h. Following two rounds of infection, the medium was replaced with induction medium (IM) lacking CHIR99021, and cells were cultured for 7–12 days with daily medium changes.

### Flow cytometry

To quantitatively analyze T expression in UiPSMs, intracellular flow cytometry was performed using an Accuri C6 Plus flow cytometer. Adherent cells were briefly washed with DPBS to remove dead or floating cells, then dissociated using 0.25% Trypsin–EDTA. Digestion was halted by adding 10% FBS, and the resulting cell suspension was centrifuged and washed twice with DPBS. Cells were fixed in 100 µl of 4% paraformaldehyde (PFA) for 20 min at room temperature, followed by two DPBS washes. For permeabilization, pre-chilled cells were gently vortexed while slowly adding cold methanol to achieve a final concentration of 90%. Cells were incubated on ice for at least 10 min and subsequently washed twice with DPBS. Next, cells were resuspended in 100 µl of blocking buffer containing primary antibody (1:100 dilution) and incubated at room temperature for 1 h. Following two additional DPBS washes, cells were resuspended in 200–500 µl of DPBS and analyzed using Accuri C6 Plus. Data were analyzed with FlowJo7.6.1 software (LLC). The primary antibody used was Milli-Mark ® Anti-Brachyury-FITC Antibody (Sigma, FCMAB302F, with a dilution of 1:100).

### Immunofluorescence

Adherent cells were fixed with 4% formaldehyde for 30 min at room temperature. Following fixation, cells were washed three times with PBS and subsequently permeabilized and blocked for 1 h at room temperature in a buffer containing PBS, 0.1% Triton X-100, and 3% BSA. Cells were then incubated overnight at 4 °C with the primary antibody diluted in the blocking buffer. After three additional PBS washes, cells were incubated with appropriate secondary antibody diluted in blocking buffer for 1 h at room temperature. Nuclear counterstaining was performed using DAPI (diluted 1:5000 in PBS) for 10 min. Prior to fluorescence microscopy, cells were washed twice more. The antibody used for immunostaining were as follows: Anti-CDX2 antibody (abcam,ab76541, with a dilution of 1:200), Anti- active β-catenin antibody (MERCK, 005–665, with a dilution of 1:100), Anti-SALL4 antibody (abcam,ab57577, with a dilution of 1:200), Anti-SOX2 antibody (Cell Signaling Technology, 3579, with a dilution of 1:200), Anti-T antibody (R&D systems, AF2085, with a dilution of 1:200), Anti-TBX6 antibody (R&D systems, AF4744, with a dilution of 1:200), Anti-CD13 antibody (abcam, ab108310, with a dilution of 1:200), Anti- β-catenin antibody (HUBIO, EM0306, with a dilution of 1:200), PHALLOIDIN (Beyotime, C2205S, with a dilution of 1:500).

### Western blot

Following dissociation and counting, 1 × 10⁶ cells were harvested and lysed on ice for 5 min in 100 μl of RIPA lysis buffer containing a protease inhibitor cocktail (Roche). Subsequently, the lysates were boiled at 100 °C for 10 min in protein loading buffer. Subsequently, the prepared samples were separated by 10%–12.5% SDS–PAGE. After separation, the proteins were transferred onto a polyvinylidene difluoride (PVDF) membrane (Millipore) using a wet transfer system. After the transfer, the membrane was incubated with primary antibodies and then secondary antibodies.

The primary antibody used in this study were: CDX2 Polyclonal Antibody (abcam, aab76541, with a dilution of 1:1000), Anti-β-catenin antibody (CST, 8480, with a dilution of 1:2000), Anti- GAPDH antibody (CST, 2118, with a dilution of 1:2000), Anti- PAX8 antibody (Proteintech, 110,336–1-AP, with a dilution of 1:1000), Anti- TBX6 antibody (R&D, AF4744, with a dilution of 1:1000), Anti- T antibody (Abclonal, A5078, with a dilution of 1:1000), Anti- WNT3A antibody (HUABIO, HA724116, with a dilution of 1:1000), Anti- active β-catenin antibody (MERCK, 005–665, with a dilution of 1:500).

### Quantitative RT-PCR

Total RNA was extracted using TRIzol reagent. From 2 μg of the total RNA, cDNA was reverse–transcribed with HiScript II Reverse Transcriptase (Vazyme biotech R222-01). Real-time quantitative PCR was then carried out using ChamQTM SYBR® qPCR Master Mix (Vazyme biotech Q311-02). The relative expression values were normalized to the expression of Gapdh. The sequences of primers used for RT-qPCR are listed in Supplementary Table 3.

### Gene editing in UiPSM cells

Knockout UiPSM cell lines were generated using the CRISPR/Cas9 system. Guide RNAs (gRNAs) were designed on the website crispr.mit.edu. Subsequently, these gRNAs were cloned downstream of the human U6 promoter in pXP (a derivative of pX330 engineered to confer puromycin resistance). For the knockout of CDX2, four pairs of gRNAs were specifically designed to delete all exons of the CDX2 gene. The gRNAs and linearized donor plasmids were transfected into UiPSM cells using Lipofectamine 3000 (Thermo Fisher Scientific) according to the manufacturer’s instructions. A total of 1 × 10⁶ cells were resuspended in UiPSM maintenance medium and plated in one well of a 6-well plate. After an incubation period of 8–10 h, the medium was replaced with fresh medium. Forty eight hours post transfection, the cells were subjected to puromycin selection for 3 days. Then, 1000 cells were seeded in one well of a 6-well plate to facilitate colony formation. Individual colonies were picked and evaluated through genotyping. The sequences of primers for the gRNAs are provided in Supplementary Table 2.

### RNA-seq analysis

Raw sequencing reads were quality-trimmed using TrimGalore (https://github.com/FelixKrueger/TrimGalore/blob/master/Docs/Trim_Galore_User_Guide.md) and aligned to the hg38 reference genome with RSEM (Li and Dewey 2011). Gene expression levels were quantified as raw counts using RSEM, and low-quality genes were filtered out. Normalization was performed with EDAseq (Risso et al. 2011). Differentially expressed genes were identified based on log₂ fold change. Functional enrichment analysis was conducted using clusterProfiler (Yu et al. 2012). Principal component analysis (PCA) was applied to all genes to explore variance in gene expression profiles.

### ATAC-seq analysis

Raw sequencing reads were quality-filtered using TrimGalore and aligned to the hg38 reference genome with Bowtie2 (Langmead et al. 2009). Read filtering and sorting were performed using SAMtools (Li et al. 2009), and duplicate reads were removed with Picard. Peak calling was carried out using MACS2 (Zhang et al. 2008), while deepTools (Quinlan and Hall 2010) was used to generate BigWig files and visualize peak distributions. Differential peaks were identified with BEDTools (Quinlan and Hall 2010). Principal component analysis (PCA) was conducted across all peaks to assess variation. Motif enrichment analysis was performed with HOMER (Heinz et al. 2010). BigWig files were visualized using the Integrative Genome Viewer (IGV) (Robinson et al. 2011). CUT&Tag data were processed following the same analytical framework. Peaks were categorized into three groups as previously described (Li et al. 2017a): CO (close to open), OC (open to close), and PO (permanently open). Within each group, peaks were further subdivided through hierarchical reclustering to refine classification.

### CUT&TAG analysis

The analytical framework closely followed that of ATAC-seq. Raw sequencing reads were processed for quality control using TrimGalore before being aligned to the hg38 reference genome with Bowtie2. Read filtering and sorting were performed with SAMtools, and duplicate reads were removed using Picard. Peak calling was conducted with MACS2, while deepTools was utilized to generate BigWig files and visualize peak distributions. Differential peak analysis was carried out using BEDTools, and principal component analysis (PCA) was applied to all detected peaks to evaluate variance. Motif enrichment analysis was performed with HOMER. BigWig files were visualized using the Integrative Genome Viewer (IGV) to support data interpretation.

### Statistical analysis and reproducibility

Sample sizes were determined based on preliminary data and established field conventions, as detailed in the figure legends. Cell culture wells were assigned to experimental groups using a random number table. To minimize subjective bias, outcome assessments were performed using automated ImageJ analysis software. For manual measurements, two investigators blinded to group allocation conducted independent assessments, and the mean of their values was recorded. All experiments were independently repeated at least three times (biological replicates), with each experiment comprising a minimum of three technical replicates. The definition of “n” (number of independent experiments) is explicitly provided in the figure legends. Data are presented as mean ± standard deviation. Statistical analyses were conducted using GraphPad Prism 8.0; two-tailed unpaired t-tests were used for comparisons between two groups, while one-way analysis of variance (ANOVA) was employed for comparisons among three or more groups. A *P*-value < 0.05 was considered statistically significant. Significance levels are denoted as *P < 0.05, **P < 0.01, and ***P < 0.001. Specific statistical methods, exact *P*-values, and sample sizes are detailed in the respective figure legends.

## Supplementary Information

Supplementary Material 1. Supplementary Tables 1-3.

Supplementary Material 2. Supplementary Figures 1-5

## Data Availability

The ATAC-seq, RNA-seq, and CUT&TAG-seq data generated in this study have been deposited in the GEO database under accession codes, GSE181352, GSE180555 and GSE185039. The authors declare that all data supporting the findings of this study are available within the article and its supplementary information files or from the corresponding author upon reasonable request.

## Abbreviations

NC: Untreated control
UiPSM: Urinal derived presomatic mesoderm
PSM: Presomatic mesoderm
NMP: Neuromesodermal progenitor
PSC: Pluripotent stem cell
UC: Human urinary-derived cell
EPZ: EPZ5676
CHIR: CHIR99021
GO: Gene Ontology
PCA: Principal component analysis
CO: Closed-to-open
OC: Open-to-closed
PO: Permanently open
TF: Transcription factor
