# Supplementary material for: Distinct regulation of Wnt signaling and EPZ5676 governs human UiPSM reprogramming via the CDX2/T/TBX6 core axis
Source: Cell Regen. 2026 Aug 24;15:28. doi: 10.1186/s13619-026-00302-z (PMC13500448; doi:10.1186/s13619-026-00302-z)
Supplement: Supplementary file 1 [file 13619_2026_302_MOESM2_ESM.pdf]

Figure S1

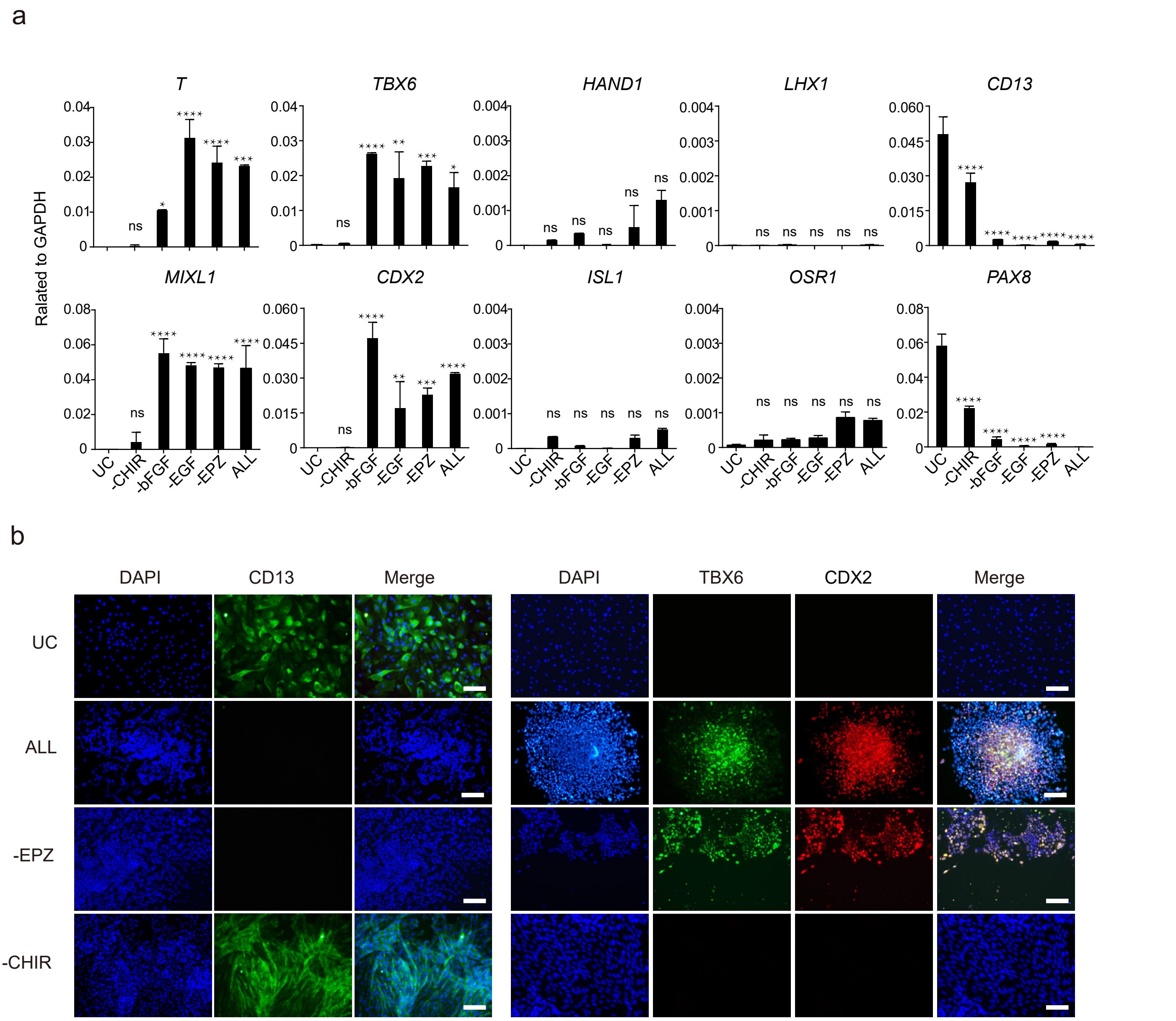

Figure S1 PSM induction determined by Wnt signaling

a. Representative gene expression of paraxial mesoderm(T,TBX6,MIXL1,CDX2) intermediate mesoderm (HAND1, ISL1), lateral plate mesoderm (LHX1, OSR1), urine cell (CD13, PAX8), pluripotency (NANOG, POU5F1) marker genes in UiPSM and HiPSM when removed each compound separately. Data are mean ± SD, n = 3 independent experiments. *p<0.05, **p<0.01, ***p < 0.001.

b. Representative immunofluorescence images showed the expression of PSM specific markers (TBX6 and CDX2) and urine cell marker gene, CD13 in UiPSM cells when dropout CHIR or EPZ. Scale bars, 100 μm.

Figure S2

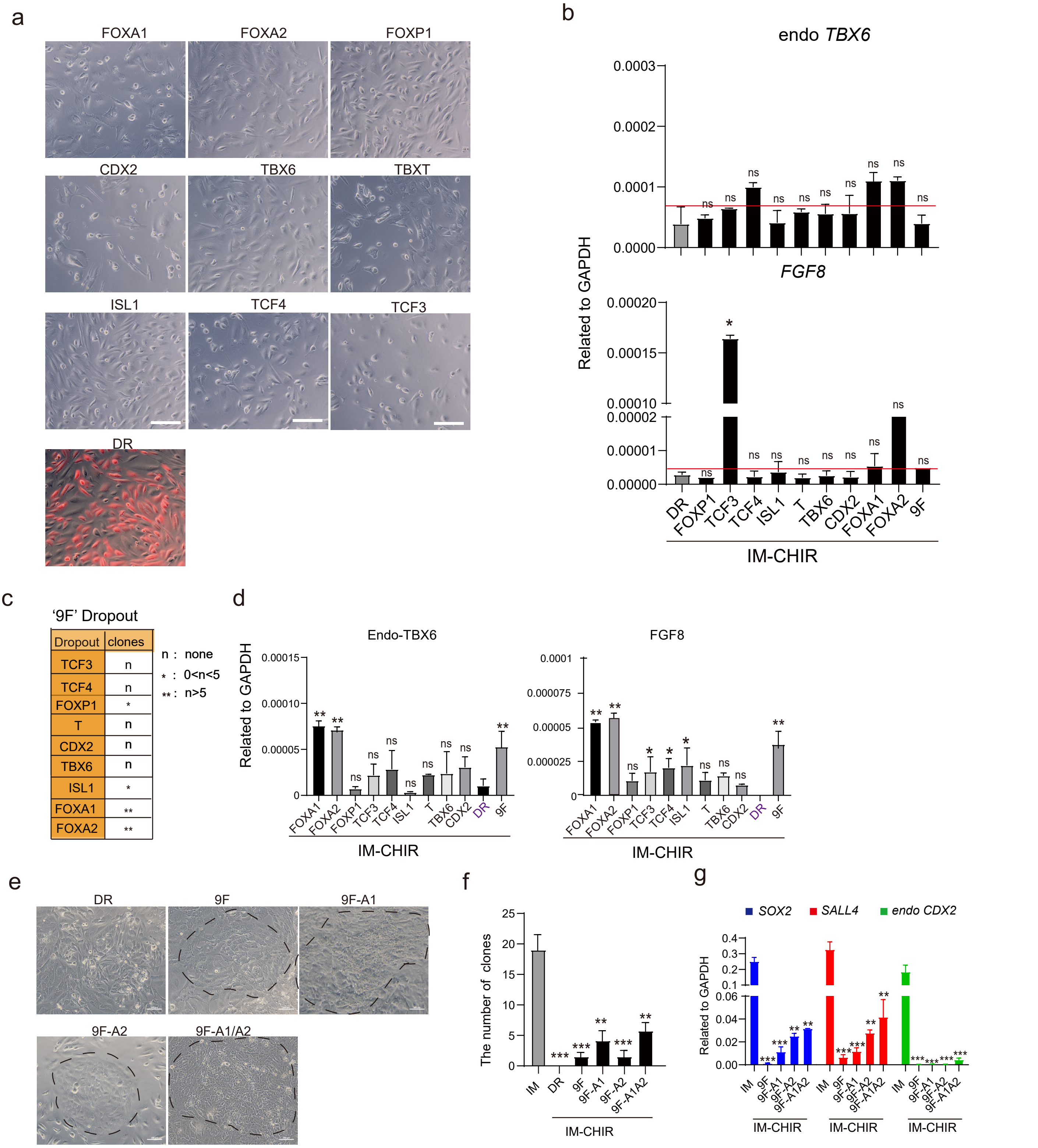

Figure S2 Downstream targets of WNT signaling

a. Representative images show the morphology of PSM clones in Urine cells with a single factor overexpression. Scale bars, 100 μm.

b. Q-PCR detected the expression of TBX6 and FGF8 in in Urine cells with single factor overexpression.

c. Statistics on the acquisition of UiPSM clones (minus CHIR) after removing a single factor from the nine factors. n, no clones; **, The number of clones is greater than 5; *, The number of clones is greater than 0 and less than 5.

d. Representative gene expression of PSM-specific genes， endogenous TBX6 and FGF8. Data are mean ± SD, n = 3 independent experiments. *p<0.05, **p<0.01, ***p < 0.001.

e. Representative images show the morphology of UiPSM clones (minus CHIR) after removing FOXA1 or/and FOXA2 from the nine factors. Scale bars, 100 μm. A1， FOXA1. A2, FOXA2.

f. Statistics on the number of PSM clones induced when overexpressing 9F and removing FOXA1 and/ or FOXA2. Data are mean ± SD, n = 3 independent experiments. *p<0.05, **p<0.01, ***p < 0.001.

g. Representative gene expression of PSM marker genes (SOX2,SALL4 and endogenous CDX2) in 9F overexpressed clones when removing FOXA1 and/ or FOXA2. Data are mean ± SD, n = 3 independent experiments. *p<0.05, **p<0.01, ***p < 0.001.

Figure S3

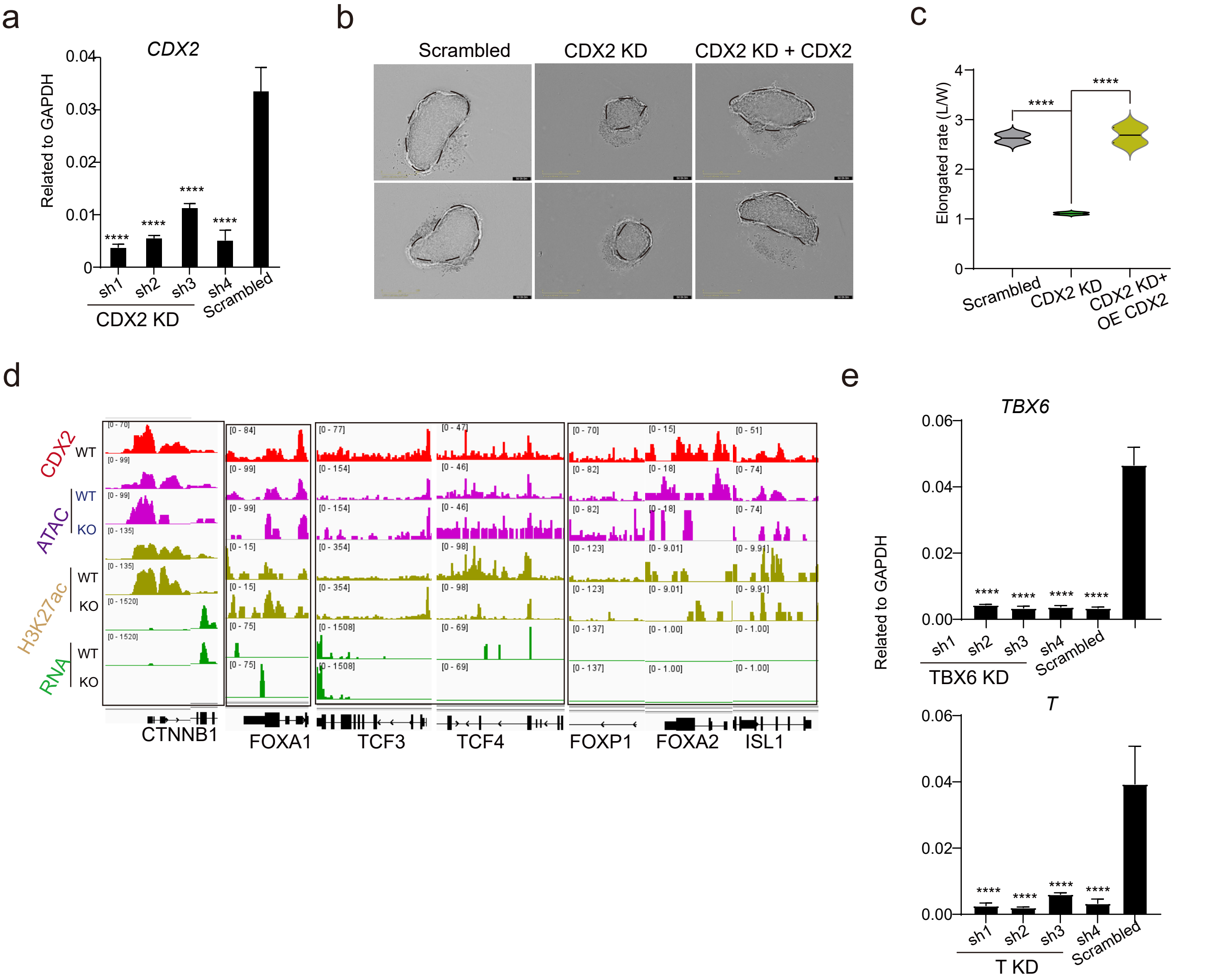

Figure S3 FGF Signaling Governs UiSomitoid Formation from UiPSM Clones

a. Bar chart showing relative mRNA expression of CDX2 normalized to GAPDH. Experimental groups: CDX2 KD clones (sh1, sh2, sh3), non-targeting scrambled control (Scrambled), and CDX2 KD with CDX2 overexpression (CDX2 KD + CDX2 OE). n=3.Statistical significance: ****P < 0.0001 (one-way ANOVA test)

b. Brightfield morphological analysis of UiSomitoids under CDX2 perturbation. Scrambled (non-targeting shRNA control), CDX2 KD, and CDX2 KD + CDX2 OE (rescuing CDX2 expression).Scale bars = 100 μm.

c. Violin plot showing the distribution of elongation rates (L/W) for individual UiSomitoids in Scrambled, CDX2 KD, and CDX2 KD + CDX2 OE groups. n=12. Statistical significance: ****P < 0.0001

d. Genome browser tracks displaying four omic layers across WT and CDX2 KO UiPSM clones for core target genes (CTNNB1, FOXA1, TCF3, FOXP1, FOXA2, ISL1)

e. qRT-PCR analysis of TBX6 and T expression following TBX6/T knockdown (KD) in UiPSM. Expression normalized to GAPDH.n=3. Statistical significance: ***P<0.001

Figure S4

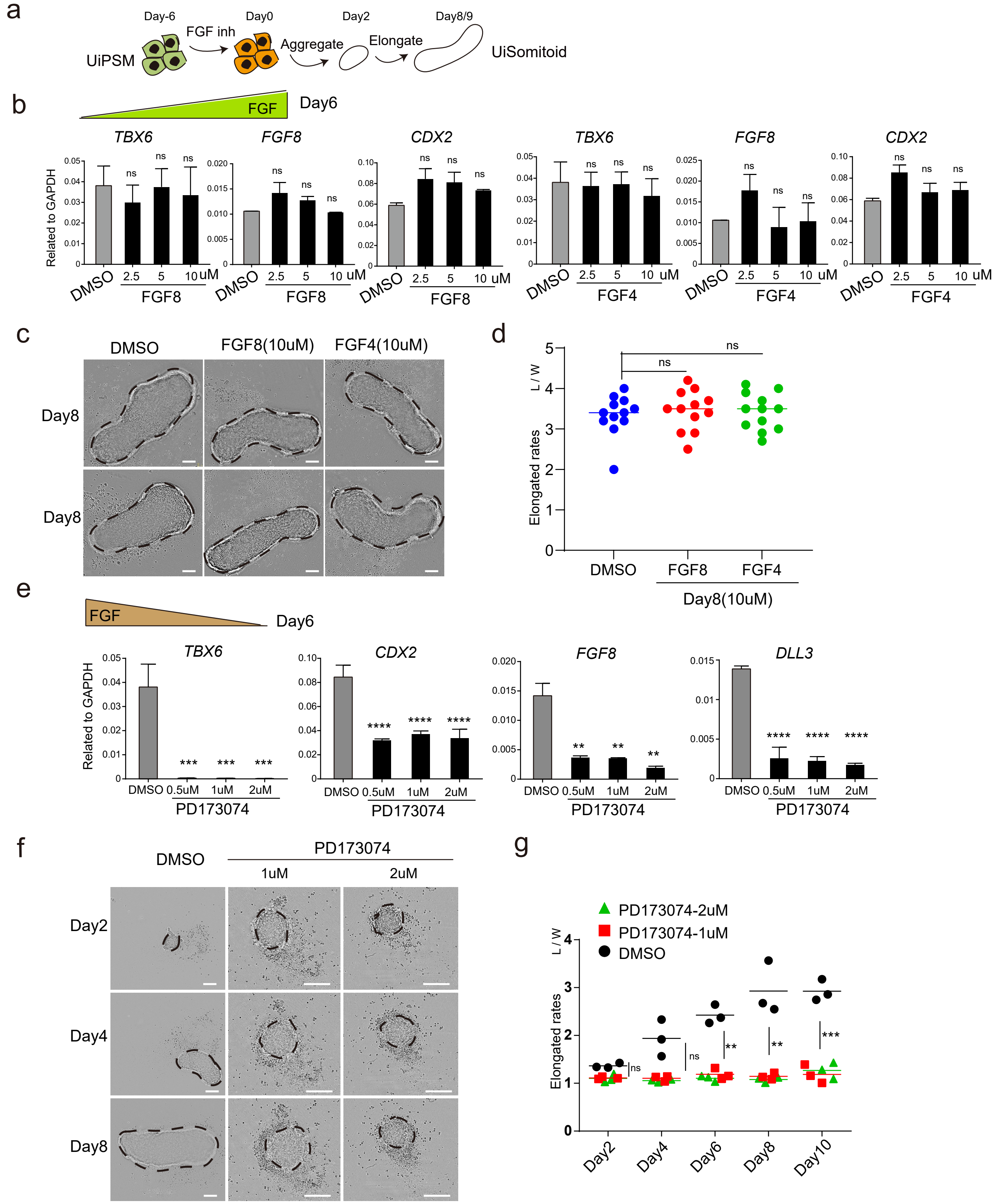

Figure S4 FGF Signaling Governs UiSomitoid Formation from UiPSM Clones

a. Schematic of UiSomitoid differentiation workflow from UiPSM clones under FGF-inhibited conditions.

b. qRT-PCR analysis of PSM genes (TBX6/CDX2) under FGF ligand dose-response (FGF8/FGF4; 2.5/5/10 μM, DMSO control) at Day6. Expression normalized to GAPDH. n=3. ns = not significant.

c. Brightfield images of Day8 UiSomitoids (DMSO/10μM FGF8/10μM FGF4). Dashed lines = UiSomitoid outlines; Scale bars = 100 μm.

d. Dot plot of UiSomitoid elongation rate (L/W) at Day8 (10μM FGF8/FGF4; DMSO control). n=12. ns = not significant.

e. qRT-PCR analysis of PSM genes (TBX6/CDX2) under FGF ligand dose-response (PD173074; 0.5/1/2 μM, DMSO control) at Day6. Expression normalized to GAPDH.n=3. **P<0.01, ***P<0.001, ****P<0.0001;ns = not significant

f. Brightfield images of Day8 UiSomitoids (DMSO and 1μM/2uM PD173074). Dashed lines = UiSomitoid outlines; Scale bars = 100 μm.

g. Dot plot of UiSomitoid elongation rate (L/W) at Day8 (1μM/2uM PD173074; DMSO control). n=3 groups(The mean was calculated from 12 samples in each group). **P<0.01, ***P<0.001. ns = not significant

Figure S5

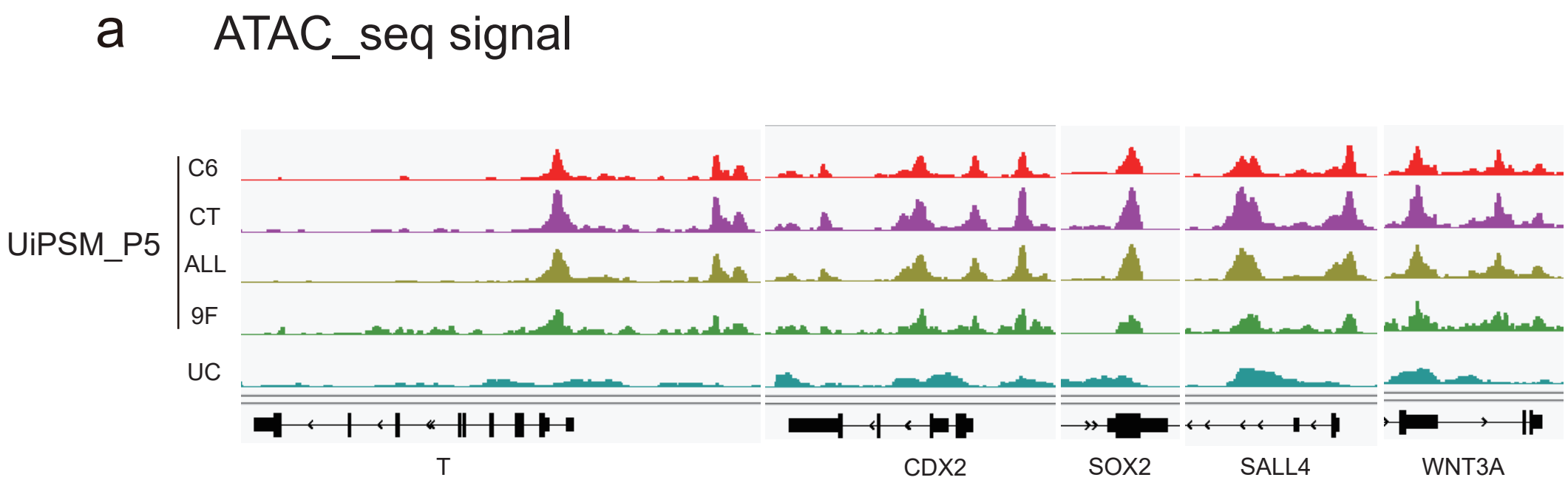

Figure S5 Characteristics of epeigenetics in UiPSM

a. ATAC-seq signal tracks showing chromatin accessibility at *T/CDX2/SOX2/SALL4/WNT3A* loci in UC and UiPSM clones (ALL) and factor induced UiPSM clones (C6/ 9F/CT) in CHIR-free condition.
