# Supplementary material for: Distinct regulation of Wnt signaling and EPZ5676 governs human UiPSM reprogramming via the CDX2/T/TBX6 core axis
Source: Cell Regen. 2026 Aug 24;15:28. doi: 10.1186/s13619-026-00302-z (PMC13500448; doi:10.1186/s13619-026-00302-z)
Supplement: Supplementary file 2 [file 13619_2026_302_MOESM1_ESM.docx]

Supplementary table 1: shRNA Primers

| Gene | Target sequence 1# | Target sequence2# |
| --- | --- | --- |
| CDX2 | ACCCACAGCCATAGACCTACA | AGACAAATATCGAGTGGTGTA |
| T(TBXT) | CGAGGAGATCACAGCTCTTAA | GACAACTCACCTGCATGTTTA |
| TBX6 | CCTCAACTTCACACCTTGATT | CAACTGAAGATTGCAGCCAAT |

Supplementary table 2: sgRNA Primers

| Gene | 5’end | 3’end |
| --- | --- | --- |
| CDX2 | aaggagccgacggagcaccg | agtagatgctgttcgtgggt |
|  | aggagccgacggagcaccgt | tcagtagatgctgttcgtgg |
|  | gctggctgcacctcagccca | catcagtagatgctgttcgt |
|  | gggctagagataaaggtagc | gcatctactgatggagattg |

Supplementary table 3: RT-PCR primers

| Gene | Forward | Reverse |
| --- | --- | --- |
| T | GCAAATCCTCATCCTCAGT | TTGTCAGAATAGGTTGGAGAAT |
| MIXL1 | AGGCAGGAGAATCACTTG | GGCTACCACAGAACCATAG |
| TBX6 | GTGACAGCCTACCAGAAC | TCGCTCCCTCTTACAGTT |
| CDX2 | TTCACTACAGTCGCTACATC | TTTCCTCTCCTTTGCTCTG |
| CD13 | TTCAGCACATCAGGCAAT | TTCTCTTCGTCGTAGTTCAC |
| PAX8 | CTGTGACAATGACACTGT | ATAGGGAGGTTGAATGGT |
| MSGN1 | AGATTCTCCTGATGACCTC | CCTCTCCGTAGTAGTGTTC |
| HAND1 | GTGCGTCCTTTAATCCTCTTC | GTGAGAGCAAGCGGAAAAG |
| ISL1 | GGTGGCTTACAGGCTAAC | AGAGGACATTGATGCTACTTC |
| LHX1 | ATTAGTGTTGTCTCAGAGTTCA | TGCGAAGGTAGAAGAGTTG |
| OSR1 | CAGCTCACCAACTACTCCTTCCTTCA | TGCAACGCGCTGAAACCATACA |
| SALL4 | CCATCACAGGTTCACATACT | TTCTAACTTCGTAGCCTTCAT |
| Endo-T | CACACTCTTAATCTTCTTCCTT | CCTGCTGTCCTCAACTAT |
| Endo-TBX6 | TGCAGGCCACACCAGTCT | CTGAGCATGGATGGCTTTTGAGT |
| Endo-CDX2 | GAGTTTGTGTTGTTGTTG | CTTCACCATATCACTTCTC |
| SOX2 | CCCAGCAGACTTCACATGT | CCTCCCATTTCCCTCGTTTT |
| MESP2 | GTCTCTCTGTGTCTCCAG | CAGTCTCTGGCATGATGG |
| WNT3A | CTGCGAGGTGAAGACATG | TGTCGTACTTGTCCTTGAG |
| WNT5A | AATGTCTTCCAAGTTCTTCCTA | ATACCTAGCGACCACCAA |
| WNT8A | TCAAGTGTGACCAGTGTA | TTGCTTCTATTCTCCTGATTC |
| FGF8 | TCTACATCTGCATGAACAAG | CACAATCTCCGTGAAGAC |
| DLL3 | GACCCTCAGCGCTACCTTTT | TACATCTTCAGGGCGATTCC |
